# TSLP Exacerbates Septic Inflammation via Murine Double Minute 2 (MDM2) Signaling Pathway

**DOI:** 10.3390/jcm8091350

**Published:** 2019-09-01

**Authors:** Na-Ra Han, Phil-Dong Moon, Hyung-Min Kim, Hyun-Ja Jeong

**Affiliations:** 1 Department of Pharmacology, College of Korean Medicine, Kyung Hee University, Seoul 02447, Korea; 2 Center for Converging Humanities, Kyung Hee University, Seoul 02447, Korea; 3 Department of Food Science & Technology, Inflammatory Diseases Research Center, Hoseo University, Asan, Chungnam 31499, Korea

**Keywords:** thymic stromal lymphopoietin, sepsis, lipopolysaccharides, macrophages, cisplatin

## Abstract

Thymic stromal lymphopoietin (TSLP) is crucial for Th2-mediated inflammation. Sepsis is a serious systemic inflammatory reaction with organ dysfunction by infection. However, the function of TSLP during sepsis is poorly understood. Thus, we investigated a role and regulatory mechanism of TSLP during sepsis. Sepsis was induced by lipopolysaccharides (LPS) or *Escherichia coli* DH5α injection in mice. TSLP levels were measured in human subjects, mice, and macrophages. TSLP deficiency or murine double minute 2 (MDM2) deficiency was induced using siRNA or an MDM2 inhibitor, nutlin-3a. We found that TSLP levels were elevated in serum of patients and mice with sepsis. TSLP deficiency lowered liver damage and inflammatory cytokine levels in mice with sepsis. TSLP was produced by the MDM2/NF-κB signaling pathway in LPS-stimulated macrophages. TSLP downregulation by an MDM2 inhibitor, nutlin-3a, alleviated clinical symptoms and septic inflammatory responses. Pharmacological inhibition of TSLP level by cisplatin reduced the septic inflammatory responses. Altogether, the present results show that TSLP exacerbates septic inflammation via the MDM2 signaling pathway, suggesting that TSLP may be a potential target for the treatment of sepsis.

## 1. Introduction

Sepsis is a systemic inflammatory reaction syndrome with infection and is a major source of morbidity and mortality [1]. Approximately 95% of sepsis cases were caused by bacterial infection and 62.2% of these were from Gram-negative bacteria with *Escherichia coli* (*E. coli*) which is responsible for about 16% according to the European Prevalence of Infection in their Intensive Care study [2]. Macrophages play a critical role in orchestrating the host immune response during sepsis [3]. Gram-negative bacterial endotoxin, lipopolysaccharide (LPS) is recognized by toll-like receptor 4 (TLR4) on macrophages and implicated in the pathogenesis of sepsis [4]. 

Thymic stromal lymphopoietin (TSLP) is produced by various cell types and is known to mainly promote allergic and inflammatory diseases [5]. TSLP activates hematopoietic cell populations expressing functional TSLP receptor (TSLPR) including both the TSLPR (cytokine receptor like factor 2, CRLF2) subunit and IL-7Rα chain (CD127) [5]. Recently, TSLP was reported to be involved in infection [6,7,8,9,10]. The increased expression levels of TSLPR and IL-7Rα in monocytes isolated from patients with Gram-negative sepsis were observed compared with healthy control subjects [7]. TSLP blockade enhanced survival in mice with sepsis [8]. The TSLP blockade suppressed the progression of chronic liver infection [9]. However, TSLP was controversially reported to exert antimicrobial activities [6], improve survival, and reduce inflammation in mice with sepsis [10]. The specific functions and mechanisms of TSLP in sepsis remain unclear because of these inconsistent findings. Despite the importance of TSLP regarding infection, the role of macrophage-derived TSLP is unknown. Thus, additional investigation is urgently needed to understand the roles and mechanisms underlying induction of TSLP during sepsis. 

Murine double minute 2 (MDM2) is well known to limit p53-mediated cell cycle arrest and apoptosis and to be a potential therapeutic target in cancer therapy [11]. However, MDM2 acts as a transcription factor to directly activate nuclear factor-κB (NF-κB), p53-independently [12]. MDM2 downregulation had potent anti-inflammatory effects in tissue damage [13]. In addition, we found that the TSLP level is regulated via MDM2 in mast cells [14]. MDM2 is a positive activator of hypoxia-inducible factor-1α (HIF-1α) [15]. LPS induces HIF-1α activation through NF-κB activation in macrophages under normoxic conditions, which is a crucial pathway in mediating LPS-induced inflammation [16]. 

In this study, we investigated a regulatory mechanism of TSLP in sepsis using LPS-stimulated macrophages and an LPS-induced sepsis model. Our findings show that TSLP is produced through the MDM2 signaling pathway in LPS-stimulated macrophages and TSLP causes organ dysfunction and an overwhelming systemic inflammatory reaction in septic mice. 

## 2. Materials and Methods

### 2.1. Human

Human study was approved by the Bioethics Committee of Kyung Hee University (KHSIRB-14-013(EA)). The Biospecimens and data used in this study were provided by the Biobank of Gyeongsang National University Hospital, a member of the Korea Biobank Network. All samples derived from the National Biobank of Korea were obtained with informed consent under institutional review board-approved protocols. All subjects provided written informed consent under the Helsinki Declaration. The biospecimens were taken from healthy volunteers (*n* = 20; 10 males, 10 females) and patients with sepsis (*n* = 30; 20 males, 10 females). The patient samples were taken within 24 h after sepsis diagnosis. The patient characteristics are listed in Supplementary Table S1.

### 2.2. Mice

Male C57BL/6 mice of 7–9 weeks old were obtained from Dae-Han Experimental Animal Center (Eumsung, Republic of Korea). TSLP^−/−^ mice on a C57BL/6 genetic background were obtained from the KOMP Repository (Grant #5U01U01HG004085, California, CA, USA). The homozygous TSLP^−/−^ was identified by reverse transcription PCR. All experiments were conducted in accordance with internationally accepted principles for laboratory animal use and care, as found in the United States guidelines (NIH publication no. 85-23, revised in 1985) and approved by the Animal Care Committee of Kyung Hee University (No. KHUASP(SE)-14-023). Sepsis was induced in male C57BL/6 mice (7–9 weeks old) by intraperitoneal injection of LPS (from *E. coli* 0111:B4, 10 mg/kg, Sigma-Aldrich Co., St. Louis, MO, USA) or *E. coli* DH5α (1 × 10^6^ CFU). Serum from the heart was obtained 4 h after LPS or *E. coli* injection. At selected time points, peritoneal cavities were washed with Dulbecco’s Modified Eagle’s Medium (DMEM; Gibco BRL, Grand Island, NY, USA) including heparin and lavage fluid was then centrifuged. Supernatants were stored at −70 °C before evaluation of cytokines by enzyme-linked immunosorbent assay (ELISA). Tissues were obtained 12 h after intraperitoneal injection of LPS or *E. coli* according to several previously published studies [17,18]. Mice were given vehicle negative control (0.001% dimethyl sulfoxide (DMSO), 10 μL/g, i.v.) or nutlin-3a (1 μM (≈0.58 mg/kg), i.v., Sigma-Aldrich Co.) daily for two days, and given phosphate-buffered saline (PBS) or LPS (10 mg/kg, i.p.) 1 h after the last nutlin-3a injection. The dose of nutlin-3a (1 μM) was determined by referring to the study of Li et al. [19]. Mice were injected i.v. with vehicle negative control (PBS) or cisplatin (100 μg/kg, Sigma-Aldrich Co.) 1 h before an i.p. injection of PBS or LPS (10 mg/kg). The serum of nutlin-3a or cisplatin-treated mice was obtained 4 h after LPS injection. Tissues of nutlin-3a or cisplatin-treated mice were obtained 12 h after LPS injection. Survival of mice was monitored after an i.p. injection of LPS (60 mg/kg) or *E. coli* (1 × 10^8^ CFU). Mice were intravenously injected with recombinant mouse TSLP (R&D Systems, 2 μg mixed with PBS) or PBS as a control (Sigma-Aldrich Co.) by referring to the studies of Piliponsky et al. [10] and Guo et al. [20]. Serum and liver tissues were obtained 4 h after intraperitoneal injection of PBS or LPS (10 mg/kg). 

### 2.3. Cell Culture

RAW 264.7 (a murine macrophage cell line derived from BALB/c, Korean Cell Line Bank, Seoul, Republic of Korea) was cultured in DMEM with 10% fetal bovine serum (FBS) and penicillin (100 U/mL)/streptomycin (100 µg/mL) at 37 °C in a humidified atmosphere of 5% CO_2_. Thioglycolate (TG)-elicited macrophages were harvested from peritoneal lavage of male C57BL/6 mice 3–4 days after intraperitoneal injection of 2.5 mL of TG. Briefly, nonadherent cells were removed by washing with PBS. Adherent peritoneal macrophages were cultured overnight in DMEM supplemented with 10% FBS and penicillin/streptomycin. Human monocyte-like cell lines, THP-1 (TIB-202; American Type Culture Collection) and HL-60 cells (Korean Cell Line Bank) derived from blood of patients with acute monocytic/promyelocytic leukemia were cultured in Roswell Park Memorial Institute (RPMI) 1640 containing 10% FBS and penicillin/streptomycin at 37 °C in a humidified atmosphere of 5% CO_2_. To induce differentiation into macrophage-like cells, THP-1 cells were differentiated with phorbol 12-myristate 13-acetate (PMA, 100 nM, Sigma-Aldrich Co.) for 24 h. HL-60 cells were differentiated with PMA (16 nM) for 72 h. The cells were washed with PBS and rested in fresh RPMI cell culture medium (without PMA) for 24 h, as reported previously [21,22,23]. RAW 264.7 cells were treated with pyrrolidine dithiocarbamate (1 μM, an inhibitor of NF-κB, Sigma-Aldrich Co.), 3-(5′-hydroxy-methyl-2′-furyl)-1-benzylindazole (10 μM, an inhibitor of HIF-1α, Sigma-Aldrich Co.), nutlin-3a (1 μM), purified rat anti-TSLP IgG antibody (neutralizing TSLP antibody, 5 μg/mL, R&D Systems, Minneapolis, MN, USA), or cisplatin (100 ng/mL) 2 h before LPS (0.1 μg/mL) stimulation. Also 0.001% DMSO or PBS was treated as a vehicle negative control for nutlin-3a or cisplatin in LPS-unstimulated RAW 264.7 cells, respectively. 

### 2.4. Assays for Biochemical Markers of Organ Dysfunction and Systemic Inflammation

We evaluated serum, tissue homogenates, and cell-free culture supernatants (3 × 10^5^ cells) by ELISA for human and mouse TSLP (R&D Systems, Minneapolis, MN, USA), IL-6 (BD Biosciences, San Diego, CA, USA), vascular endothelial growth factor (VEGF, R&D Systems), intercellular adhesion molecule-1 (ICAM-1, R&D Systems), macrophage inflammatory protein 2 (MIP2, chemokine (C-X-C motif) ligand 2, CXCL2, R&D Systems), and tumor necrosis factor-α (TNF-α, BD Biosciences) following the manufacturer’s protocols. Total protein concentrations in tissue homogenates were determined using a bicinchoninic acid (BCA; Sigma-Aldrich Co., St. Louis, MO, USA) protein assay kit. These biochemical markers were analyzed using an ELISA microplate reader (Molecular Devices, LLC., Sunnyvale, CA, USA) at 405 nm. Aspartate aminotransferase (AST, Sigma-Aldrich Co.), alanine aminotransferase (ALT, Sigma-Aldrich Co.), blood urea nitrogen (BUN, Arbor Assays, Ann Arbor, MI, USA), and creatine kinase (CK, Abcam, Cambridge, UK) levels were measured in serum by the manufacturer’s protocols. Nitric oxide concentration in cell-free culture supernatants from RAW 264.7 cells was measured by the Griess method. Briefly, 100 μL of cell-free culture supernatant was mixed with 50 μL of 0.1% N-(1-naphtyl)-ethylenediamine dihydrochloride in distilled water and 50 μL of 1% sulfanilic acid in 5% phosphoric acid in a 96-well plate. The absorbance was measured at 540 nm using an ELISA microplate reader. 

### 2.5. PCR

RNA isolation was performed on liver, lung, kidney, and large intestine tissues, RAW 264.7 (1 × 10^6^ cells), peritoneal macrophages (1 × 10^6^ cells), THP-1 (1 × 10^6^ cells), and HL-60 (1 × 10^6^ cells) using an easy-BLUE^TM^ RNA extraction kit (iNtRON Biotech Inc., Seongnam, Korea). The isolated total RNA was dissolved in 50 μL RNase-free water. Each concentration of the total RNA was measured by NanoDrop spectrophotometry (Thermo Scientific, Worcester, MA, USA). The total RNA was incubated at 70 °C for 5 min, placed on ice, and reverse-transcribed to cDNA for 60 min at 42 °C and 5 min at 94 °C using a cDNA synthesis kit (Bioneer Corporation, Daejeon, Korea). Real-time PCR was performed with primers as in the Supplementary Table S2 using SYBR Green Master Mix and an ABI StepOne real-time PCR System (Applied Biosystems, Foster City, CA, USA). RNA was normalized to expression levels of GAPDH. Relative expression was analyzed by using ^ΔΔ^CT method. 

### 2.6. Transfection with siRNA

Mice were intravenously injected with scramble control siRNA or TSLP siRNA (20 µM, TSLP SMARTpool, Supplementary Table S3, Dharmacon Inc., Chicago, IL, USA) + atelocollagen (Atelogene in vivo siRNA transcription kit, Cosmo Bio Co., LTD, Japan) mixture (final concentration: 10 µM) with reference to the instructions of manufacturer and reports of Tasaki et al. [24] and Takeshita et al. [25]. Mice received TSLP siRNA 24 h before LPS or PBS injection according to several previously published studies [26,27]. TSLP silencing was tested in tissues obtained 12 h after PBS or LPS injection. We used Lipofectamine™ 2000 (Invitrogen, Carlsbad, CA, USA) to transiently transfect siRNA (HIF-1α (20 nM), MDM2 (20 nM), and TSLP (20 nM) SMARTpool, Dharmacon Inc.) into RAW 264.7 cells with reference to a report of Zheng et al. [28]. At 48 h after transfection with HIF-1α siRNA, MDM2 siRNA, TSLP siRNA, or scramble control siRNA, RAW 264.7 cells were stimulated with LPS (0.1 μg/mL). 

### 2.7. Immunoblot Analysis

Samples for immunoblotting were obtained from liver, lung, kidney, and large intestine tissues, RAW 264.7 cells (5 × 10^6^ cells), and peritoneal macrophages (5 × 10^6^ cells). Tissues were homogenized in homogenization buffer. The cells were lysed in cell lysis buffer (R&D Systems) containing protease inhibitor. These were subjected to separation on SDS-PAGE gels and transferred onto nitrocellulose membranes. The following antibodies were used: anti-MDM2 (Cat# sc-965, mouse monoclonal antibody, Santa Cruz Biotechnology, Santa Cruz, CA, USA), NF-κB (Cat# sc-8008, mouse monoclonal antibody, Santa Cruz Biotechnology), HIF-1α (Cat# sc-13515, mouse monoclonal antibody, Santa Cruz Biotechnology), p53 (Cat# sc-6243, rabbit polyclonal antibody, Santa Cruz Biotechnology), actin (Cat# sc-8432, mouse monoclonal antibody, Santa Cruz Biotechnology), GAPDH (Cat# sc-32233, mouse monoclonal antibody, Santa Cruz Biotechnology), and histone (Cat# sc-10806, rabbit polyclonal antibody, Santa Cruz Biotechnology) antibodies. The membranes were incubated with peroxidase-conjugated second antibodies (Santa Cruz Biotechnology). Signals were developed with an enhanced chemiluminescence solution (DoGenBio Co., Seoul, Korea). 

### 2.8. Nuclear Extract

RAW 264.7 cells were suspended in 40 μL of buffer A (10 mM HEPES/KOH, 0.1 mM EDTA, 2 mM MgCl_2_, 1 mM dithiothreitol, 10 mM KCl, and 0.5 mM phenylmethylsulfonyl fluoride) for 15 min on ice, lysed with 0.6 μL of 10% Nonidet P-40. After centrifuge, the nuclei pellets were resuspended in 20 μL of buffer A and 40 μL of buffer B (50 mM HEPES/KOH, 0.1 mM EDTA, 1 mM dithiothreitol, 50 mM KCl, 300 mM NaCl, 10% glycerol, and 0.5 mM phenylmethylsulfonyl fluoride) for 20 min on ice. After centrifuge, the supernatant (nuclear extract) was used to analyze expression levels of NF-κB, HIF-1α, and histone. 

### 2.9. MTT Assay

Cell viability was analyzed by a 3-(4,5-dimethylthiazol-2-yl)-2,5-diphenyltetrazolium bromide (MTT, Sigma-Aldrich Co.) assay. For this assay, RAW 264.7 cells (1 × 10^4^) were plated on a 96-well plate and subjected to LPS, *E. coli*, nutlin-3a, or cisplatin for 24 h. MTT reagent (5 mg/mL) was added to each well and incubated at 37 °C for 4 h. The insoluble formazan product was dissolved in dimethyl sulfoxide. Optical density was analyzed with an ELISA microplate reader at 540 nm.

### 2.10. HIF-1α Luciferase Assay

RAW 264.7 cells were transfected with luciferase reporter plasmid combined with a HIF-1α-Luc reporter for 48 h, treated with nutlin-3a for 2 h, stimulated with LPS for 48 h, and then lysed, followed by analysis of the reporter activity using a luminometer 1420 luminescence counter (Perkin Elmer, Waltham, MA, USA). The relative luciferase activity was determined by the ratio of *firefly* luciferase activity to *renilla* luciferase activity.

### 2.11. Statistics

Pairwise comparisons were made using independent *t*-test, while multiple comparisons were made using one-way ANOVA analysis with Tukey’s post hoc test using IBM SPSS statistics 23 (IBM Corp., Armonk, NY, USA). A *p* value of less than 0.05 was considered statistically significant. Data are presented as the mean ± standard error of the mean (SEM). Pilot experiments were performed to estimate the sample size (*n* = 10 mice/group, Type I error 0.05, power 96.31%). In vitro data are representative of three independent experiments (*n* = 5/group). 

## 3. Results

### 3.1. TSLP Is Associated with Sepsis

**Figure 1:**
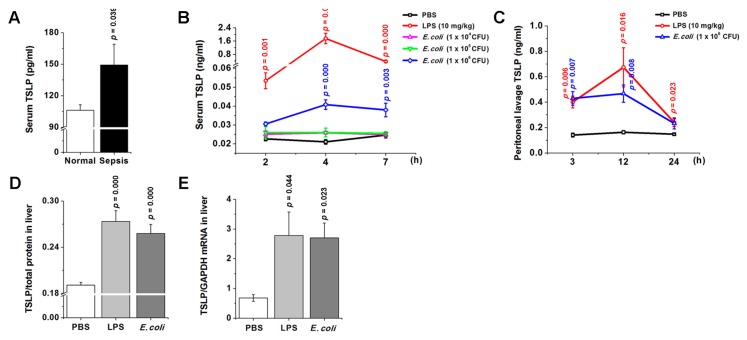
TSLP is associated with sepsis. (**A**) TSLP levels in serum of patients with sepsis were analyzed by ELISA. Normal (Healthy volunteers, *n* = 20); Sepsis (Patients with sepsis, *n* = 30). A *p* value indicates the significant difference between normal and sepsis (**B**) TSLP levels were analyzed in serum of mice following LPS or *E. coli* injection by ELISA. (**C**) TSLP levels were analyzed in peritoneal lavage of mice following LPS or *E. coli* injection by ELISA. (**D**) TSLP protein levels were analyzed 12 h after LPS (10 mg/kg) or *E. coli* (1 × 10^6^ CFU) injection by ELISA. (**E**) TSLP mRNA expression was analyzed 12 h after LPS (10 mg/kg) or *E. coli* (1 × 10^6^ CFU) injection by real-time PCR (*n* = 10/group). A *p* value indicates the significant difference between PBS and LPS. PBS, phosphate-buffered saline; LPS, lipopolysaccharide; TSLP, thymic stromal lymphopoietin.

We first measured the TSLP level in serum of human patients with sepsis. As shown in Figure 1A, the TSLP level significantly increased in serum of patients with sepsis (*p* < 0.05). However, there were no significant differences in the TSLP levels between males and females, older and younger patients, patients who died and patients who survived, or patients with a specific bacterial species and others. We performed a time-course study to determine TSLP levels in serum and peritoneal lavage of mice following LPS or *E. coli* injection according to previous reports [29,30,31]. The results shown in Figure 1B revealed a significant increase in TSLP level in serum around 2 h after LPS or *E. coli* injection and reached a maximum at around 4 h after the injection (*p* < 0.05). TSLP level began to decline gradually around 4 h after the injection. At the same time, the serum IL-6 and TNF-α levels which are early biomarkers for sepsis [32] also reached a maximum at around 4 h and 2 h after the injection, respectively (*p* < 0.05; Supplementary Figure S1A), indicating that TSLP may be an early biomarker for sepsis. TSLP level in peritoneal lavage also significantly increased after LPS or *E. coli* injection (Figure 1C). Next, we investigated whether TSLP is also associated with organ dysfunction during sepsis. TSLP expression significantly increased in liver at both protein and mRNA levels after LPS or *E. coli* injection as compared with PBS-injected controls (*p* < 0.05; Figure 1D,E). Of course, the protein and mRNA expressions of TSLP significantly increased in lung, kidney, and large intestine after LPS injection (*p* < 0.05; Supplementary Figure S1B–D).

### 3.2. Systemic Inflammatory Reaction Is Blunted in the Absence of TSLP during Sepsis

**Figure 2:**
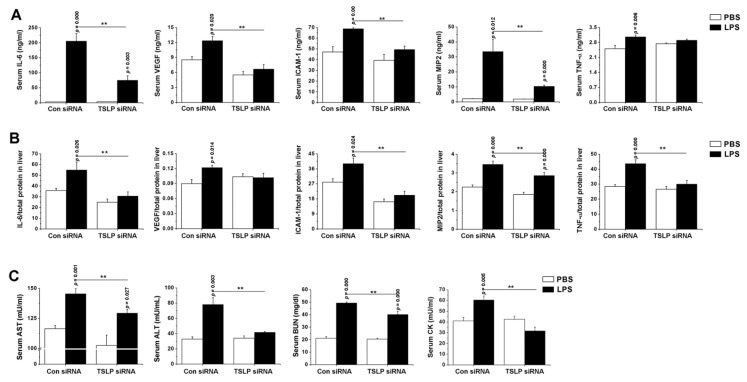
Systemic inflammatory reaction is blunted by the deficiency of TSLP during sepsis. Mice were given either scramble control or TSLP-specific siRNA + atelocollagen mixture. Each level in (**A**) serum at 4 h and (**B**) liver homogenate at 12 h following LPS (10 mg/kg) injection was analyzed by ELISA. Adducts were normalized to total protein in liver homogenate. (**C**) Each level was analyzed in serum (*n* = 10/group). A *p* value indicates the significant difference between PBS and LPS. ** *p* < 0.05 vs. Con siRNA-received and LPS-injected control mice. PBS, phosphate-buffered saline; LPS, lipopolysaccharide; TSLP, thymic stromal lymphopoietin; Con, control; siRNA, small interfering RNA; VEGF, vascular endothelial growth factor; ICAM-1, intercellular adhesion molecule-1; MIP2, macrophage inflammatory protein 2; AST, aspartate aminotransferase; ALT, alanine aminotransferase, BUN, blood urea nitrogen; CK, creatine kinase.

To assess whether TSLP contributes to the development of systemic inflammatory reaction during sepsis, we analyzed dynamic changes of inflammatory cytokine levels in TSLP-deficient mice. First, we tested the reduction of TSLP levels in tissues of TSLP siRNA-received mice. Since the reduced TSLP levels in liver existed for 36 h or 48 h after TSLP siRNA injection (*p* < 0.05; Supplementary Figure S2A), we confirmed the reduction of TSLP levels in the liver, lung, kidney, and large intestine injected with PBS or LPS 36 h after TSLP siRNA injection (*p* < 0.05; Supplementary Figure S2B,C). We found that levels of IL-6, VEGF, ICAM-1, and MIP2 were significantly lower in the serum of TSLP-deficient mice than those of control mice 12 h after LPS injection (*p* < 0.05; Figure 2A). Furthermore, TSLP-deficient mice had significantly less IL-6, ICAM-1, MIP2, and TNF-α levels in liver (*p* < 0.05; Figure 2B). Also, similar significant differences in the inflammatory cytokines were observed in lung, kidney, and large intestine between both groups after LPS injection (*p* < 0.05; Supplementary Figure S2D–F). In addition, TSLP deficiency significantly lowered serum AST, ALT, BUN, and CK levels relative to control mice after LPS injection (*p* < 0.05; Figure 2C). We validated the significant differences in the inflammatory cytokines in serum and liver by using TSLP^−/−^ mice (*p* < 0.05; Supplementary Figure S3A,B). Furthermore, we examined whether the inflammatory cytokines are also reduced in lung of TSLP^−/−^ mice because alveolar macrophages are present in the lung and might play a role in TSLP stimulation. As expected, the inflammatory cytokines significantly decreased in the lung of TSLP^−/−^ mice (*p* < 0.05; Supplementary Figure S3C). We observed that LPS-injected TSLP^−/−^ mice had a significantly higher survival than LPS-injected wild-type mice (*p* < 0.05; Supplementary Figure S3D).

### 3.3. TSLP Causes Systemic Inflammatory Reaction and Organ Dysfunction in Septic Mice

We further explored a potential role of TSLP underlying pathogenesis of sepsis by injecting recombinant TSLP into septic mice (TSLP plus LPS-injected mice). IL-6 and AST levels significantly increased in serum of TSLP plus LPS-injected septic mice vs. LPS-injected septic mice (Supplementary Figure S4A,B; *p* < 0.05), suggesting a synergistic effect of TSLP on LPS-induced septic responses. However, the TSLP plus LPS-injected septic mice did not show significant changes in VEGF, ICAM-1, MIP2, and TNF-α levels. ALT levels were not significantly different between TSLP plus LPS-injected septic mice and LPS-injected septic mice (Supplementary Figure S4C). BUN level slightly increased in the serum of TSLP plus LPS-injected septic mice vs. LPS-injected septic mice (Supplementary Figure S4D). Taken together, the findings suggest that TSLP may be closely linked to a synergistic effect on IL-6 and AST levels during sepsis. 

### 3.4. TSLP Production Is Mediated by NF-κB and HIF-1α in Macrophages 

**Figure 3:**
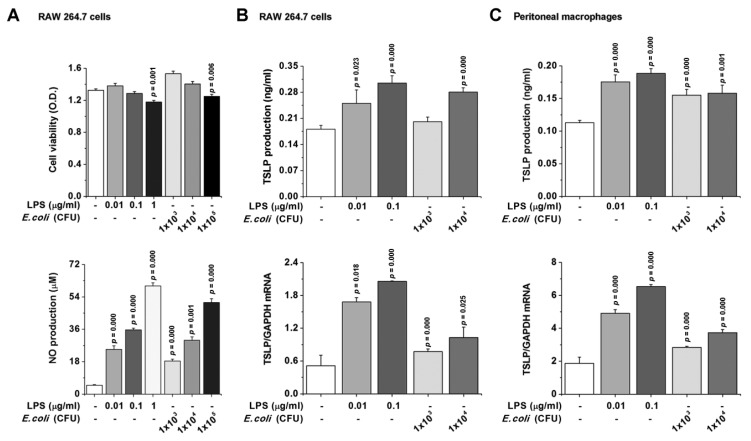
TSLP is produced in macrophages. (**A**) RAW 264.7 cells were stimulated with LPS or *E. coli* for 24 h. (**A**, upper panel) Cell viability was analyzed by an MTT assay. (**A**, lower panel) NO concentration was measured by the Griess method. (**B**,**C**) Cells were stimulated with LPS or *E. coli* (upper panel) for 24 h for ELISA or (lower panel) 8 h for real-time PCR. Data are representative of three independent experiments (*n* = 5/group). A *p* value indicates the significant difference between PBS and LPS. LPS, lipopolysaccharide; TSLP, thymic stromal lymphopoietin; NO, nitric oxide.

We next investigated whether TSLP is involved in sepsis in a TLR4-dependent manner and thus focused on biological consequences of TSLP induced by LPS or *E. coli* in macrophages. First, the dose of LPS or *E. coli* was determined in RAW 264.7 cells by an MTT assay (Figure 3A, upper panel). As a complement to viability, we checked activation (an increase in nitric oxide (NO) production) after LPS or *E. coli* stimulation in RAW 264.7 cells (Figure 3A, lower panel). LPS or *E. coli* stimulation significantly increased the production (Figure 3B, upper panel) and mRNA expression (Figure 3B, lower panel) of TSLP in RAW 264.7 cells (*p* < 0.05). Also, LPS or *E. coli* significantly induced increases in the production (Figure 3C, upper panel) and mRNA expression (Figure 3C, lower panel) of TSLP in peritoneal macrophages (*p* < 0.05). Furthermore, we confirmed that the findings obtained from mouse macrophages also occur in human by using THP-1 and HL-60 cells, which are commonly used as surrogates of monocytes isolated from human peripheral blood mononuclear cells [33,34,35]. Expectedly, LPS or *E. coli* significantly increased the production and mRNA expression of TSLP in PMA-differentiated THP-1 and HL-60 macrophage-like cells (*p* < 0.05; Supplementary Figure S5). Importantly, macrophage-derived TSLP production was found to be dependent on dose of LPS or *E. coli.* In the next series of studies, we sought to assess how TSLP is produced in macrophages. As shown in Supplementary Figure S6A, TSLP production was significantly downregulated via inhibitions of NF-κB and HIF-1α (*p* < 0.05). To directly show that HIF-1α potentiates TSLP production from macrophages, we performed HIF-1α siRNA silencing experiments. After LPS stimulation, we confirmed HIF-1α mRNA reduction using HIF-1α siRNA by real-time PCR (*p* < 0.05; Supplementary Figure S6B). The production (*p* < 0.05) and mRNA expression of TSLP in HIF-1α siRNA-transfected RAW 264.7 cells were downregulated as compared with those of control siRNA-transfected RAW 264.7 cells after LPS stimulation (Supplementary Figure S6C), indicating that HIF-1α reduction allows TSLP downregulation. Impaired inflammatory reactions in HIF-1α siRNA-transfected RAW 264.7 cells were further confirmed by decreased VEGF, ICAM-1, and TNF-α production (*p* < 0.05; Supplementary Figure S6D). 

### 3.5. TSLP Is Produced via MDM2 Signaling in Macrophages

**Figure 4:**
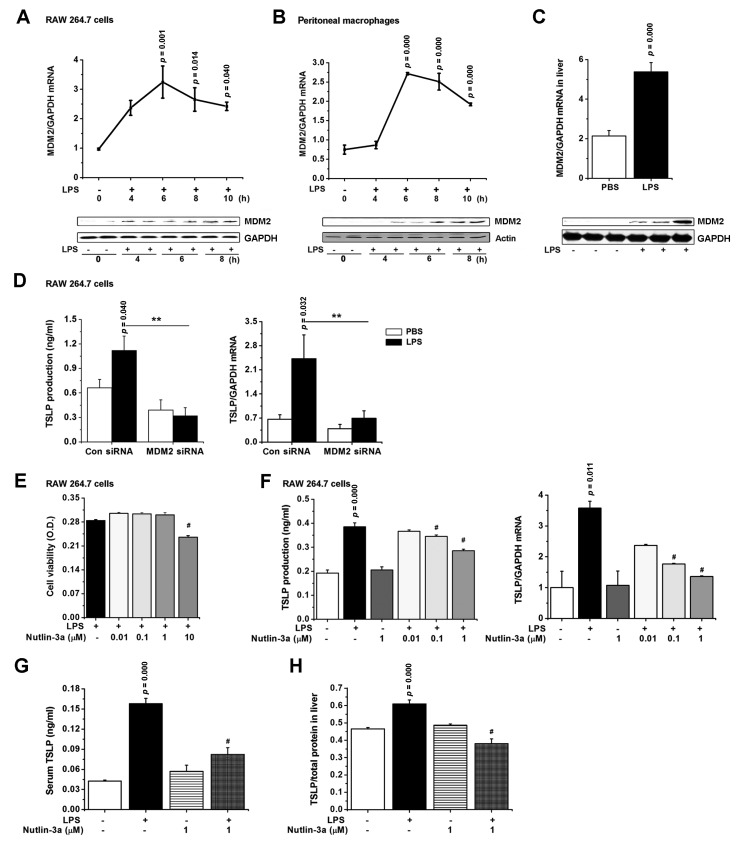
TSLP is produced via MDM2 signaling in macrophages. (**A**,**B** upper panel) The mRNA and (**A**,**B** lower panel) protein expressions of MDM2 were analyzed by real-time PCR and Western blot. (**C**, upper panel) The mRNA and (**C**, lower panel) protein expressions of MDM2 in liver were analyzed by real-time PCR and Western blot. (**D**, left) The production of TSLP 24 h and (**D**, right) mRNA expression of TSLP 8 h after LPS stimulation were analyzed in RAW 264.7 cells by ELISA and real-time PCR. ** *p* < 0.05 vs. Con siRNA transfection and LPS stimulation. (**E**) Cell viability was analyzed by an MTT assay. RAW 264.7 cells were treated with nutlin-3a for 2 h and stimulated with LPS (**F**, left) for 24 h for ELISA and (**F**, right) 8 h for real-time PCR. Data are representative of three independent experiments (*n* = 5/group). # *p* < 0.05 vs. LPS stimulation. (**G**) Serum at 4 h and (**H**) liver homogenate at 12 h following LPS injection from nutlin-3a-treated septic mice were subjected to ELISA (*n* = 10/group). Adducts were normalized to total protein in liver homogenate. 0.001% DMSO was treated as a vehicle negative control for nutlin-3a in LPS-unstimulated group. A *p* value indicates the significant difference between PBS and LPS. # *p* < 0.05 vs. LPS-injected mice. PBS, phosphate-buffered saline; LPS, lipopolysaccharide; TSLP, thymic stromal lymphopoietin; Con, control; siRNA, small interfering RNA; MDM2, murine double minute 2.

In line with the reports showing that MDM2 is a positive activator of NF-κB [12] and HIF-1α [15], MDM2 mRNA expression slightly increased 4 h after LPS stimulation and reached a maximum at around 6 h in RAW 264.7 cells (*p* < 0.05; Figure 4A, upper panel). MDM2 mRNA expression began to decline after at around 6 h and further declined at 10 h in RAW 264.7 cells (*p* < 0.05; Figure 4A, upper panel). MDM2 protein expression reached a maximum at around 8 h after LPS stimulation in RAW 264.7 cells (Figure 4A, lower panel). These results from RAW 264.7 cells were consistent with the results that were obtained from peritoneal macrophages (*p* < 0.05; Figure 4B). The mRNA (*p* < 0.05) and protein expressions of MDM2 increased in liver (Figure 4C), lung, kidney, and large intestine (Supplementary Figure S7) of LPS-injected septic mice. To directly show that MDM2 mediates TSLP production, we performed MDM2 siRNA silencing experiments in macrophages and studied its response to LPS. We confirmed that MDM2 mRNA expression was significantly inhibited in MDM2 siRNA-transfected RAW 264.7 cells by real-time PCR (*p* < 0.05; Supplementary Figure S8A). MDM2 siRNA-transfected RAW 264.7 cells showed significant decreases in production and mRNA expression of TSLP as compared with controls after LPS stimulation (*p* < 0.05; Figure 4D), indicating that MDM2 mediates TSLP production in macrophages. In addition, MDM2 siRNA-transfected RAW 264.7 cells showed impaired ability to produce IL-6 (*p* < 0.05), VEGF (*p* < 0.05), ICAM-1 (*p* < 0.05), and TNF-α (Supplementary Figure S8B) as well as TSLP. To further address a putative functional contribution of MDM2 to TSLP production, we treated an MDM2 inhibitor, nutlin-3a in RAW 264.7 cells. First, we determined doses of nutlin-3a that did not show cytotoxicity in RAW 264.7 cells by referring to the study of Li et al. [19] (Figure 4E). Nutlin-3a significantly decreased the production and mRNA expression of TSLP in LPS-stimulated RAW 264.7 cells (*p* < 0.05; Figure 4F). Nutlin-3a markedly decreased TSLP mRNA expression, but slightly decreased serum TSLP levels 24 h after LPS injection (*p* < 0.05; Supplementary Figure S9A), providing that nutlin-3a affects transcription rather than translation. In addition, nutlin-3a significantly inhibited serum TSLP levels 4 h after LPS injection (*p* < 0.05; Figure 4G). Nutlin-3a significantly reduced TSLP levels in liver and lung of mice with sepsis (*p* < 0.05; Figure 4H and Supplementary Figure S9B). Thus, these findings suggest that MDM2 would be a critical factor in TSLP level during sepsis.

### 3.6. Nutlin-3a, an MDM2 Inhibitor, Regulates Inflammatory Responses during Sepsis

We investigated whether nutlin-3a could downregulate inflammatory responses during sepsis. Nutlin-3a significantly suppressed production of IL-6, VEGF, ICAM-1, MIP2, and TNF-α in LPS-stimulated RAW 264.7 cells (*p* < 0.05; Supplementary Figure S10A). Next, we examined the mechanism of nutlin-3a for regulating the production of inflammatory cytokines in RAW 264.7 cells. Nutlin-3a dose-dependently decreased expression of NF-κB and HIF-1α, and increased expression of p53 in LPS-stimulated RAW 264.7 cells (Supplementary Figure S10B). Nutlin-3a dose-dependently suppressed luciferase activity of HIF-1α (*p* < 0.05; Supplementary Figure S10C). In addition, nutlin-3a significantly decreased IL-6, VEGF, ICAM-1, and CK levels in serum (*p* < 0.05; Supplementary Figure S10D) and IL-6 and VEGF levels in the liver of mice with sepsis (*p* < 0.05; Supplementary Figure S10E). 

### 3.7. TSLP Upregulates Macrophages-Mediated Inflammatory Responses during Sepsis

**Figure 5:**
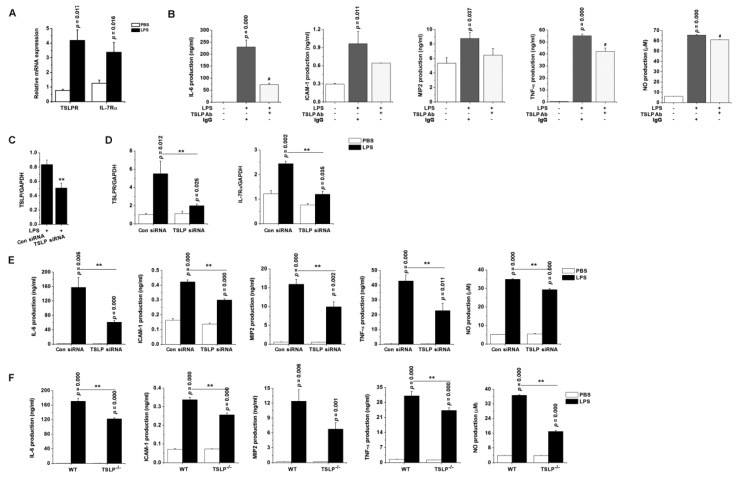
TSLP-dependent proinflammatory environment predisposes to the development of sepsis. (**A**) The mRNA expression of TSLPR and IL-7Rα were analyzed in RAW 264.7 cells by real-time PCR. (**B**) RAW 264.7 cells were stimulated with LPS (0.1 μg/mL) in the absence or presence of neutralizing anti-TSLP antibody (5 µg/mL) or nonimmune IgG (5 µg/mL) for 24 h. # *p* < 0.05 vs. LPS stimulation. RAW 264.7 cells were transfected with scramble control siRNA or TSLP-specific siRNA. After LPS stimulation, the mRNA expression levels of (**C**) TSLP, (**D**) TSLPR, and IL-7Rα were analyzed in the transfected cells by real-time PCR. (**E**) The transfected RAW 264.7 cells were stimulated with LPS for 24 h. ** *p* < 0.05 vs. Con siRNA transfection and LPS stimulation. (**F**) The peritoneal macrophages isolated from TSLP^−/−^ mice were stimulated ex vivo with LPS for 24 h. Each production was analyzed by ELISA. Nitric oxide concentration was measured by the Griess method. Data are representative of three independent experiments (*n* = 5/group). A *p* value indicates the significant difference between PBS and LPS. ** *p* < 0.05 vs. LPS-stimulated WT macrophages. PBS, phosphate-buffered saline; LPS, lipopolysaccharide; TSLP, thymic stromal lymphopoietin; Ab, antibody; Con, control; siRNA, small interfering RNA; ICAM-1, intercellular adhesion molecule-1; MIP2, macrophage inflammatory protein 2; NO, nitric oxide; WT, wild-type.

We next sought to understand how TSLP released from LPS-stimulated macrophages contributes to septic responses in macrophages. TSLP neutralization allowed significant decreases in production of IL-6, TNF-α, and NO after LPS stimulation in RAW 264.7 cells expressing TSLPR and IL-7Rα (*p* < 0.05; Figure 5A,B). We performed TSLP siRNA silencing experiments and studied whether TSLP affects the production of inflammatory cytokines responsive to LPS in RAW 264.7 cells. The mRNA expression of TSLPR and IL-7Rα, and production of inflammatory cytokines and NO were attenuated in TSLP siRNA-transfected cells as compared with those of controls after LPS stimulation (*p* < 0.05; Figure 5C–E). IL-6 levels were low, but not significantly reduced, in RAW 264.7 cells transfected with siRNA for both TSLP and MDM2 as compared with those of RAW 264.7 cells transfected with siRNA alone upon LPS stimulation (Supplementary Figure S11), suggesting TSLP and MDM2 may play a synergetic role in regulating LPS signaling. To provide direct evidence that a lack of TSLP undermines production of inflammatory cytokines, we stimulated TSLP^−/−^ macrophages with LPS. TSLP^−/−^ macrophages showed impaired ability to produce inflammatory cytokines by LPS stimulation as compared with wild-type macrophages ex vivo (*p* < 0.05; Figure 5F), indicating that macrophage-derived TSLP is involved in the production of inflammatory cytokines. 

### 3.8. Pharmacological Inhibition of TSLP by Cisplatin Protects Mice against Lethal Sepsis

**Figure 6:**
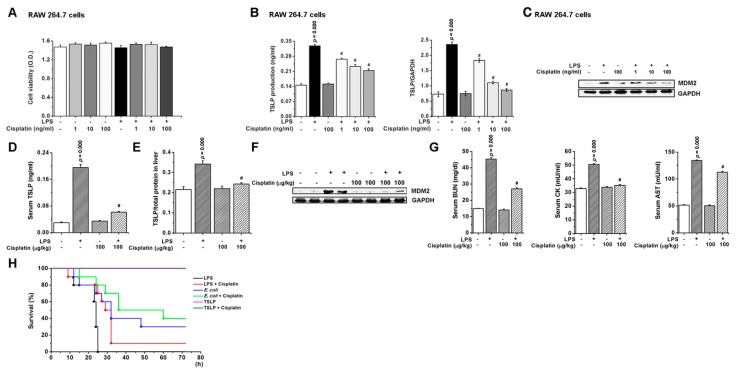
Pharmacological inhibition of TSLP by cisplatin protects mice against lethal sepsis. (**A**) RAW 264.7 cells were treated with cisplatin for 2 h, followed by incubation for 24 h with LPS (0.1 μg/mL). Cell viability was analyzed by MTT assay. RAW 264.7 cells were treated with cisplatin for 2 h, followed by incubation for (**B**, Left) 24 h with LPS (0.1 μg/mL) for analysis of TSLP production and (**B**, Right) for 8 h with LPS (0.1 μg/mL) for analysis of TSLP mRNA expression. # *p* < 0.05 vs. LPS stimulation. (**C**) RAW 264.7 cells were treated with cisplatin for 2 h, followed by incubation for 8 h with LPS (0.1 μg/mL). MDM2 expression in cells lysates was analyzed by Western blot. Each TSLP level in (**D**) serum at 4 h and (**E**) liver homogenate at 12 h following LPS (10 mg/kg) injection was analyzed by ELISA. Adducts were normalized to total protein in liver homogenate. (**F**) MDM2 expression in liver homogenate at 12 h following LPS (10 mg/kg) injection was analyzed by Western blot. For immunoblots, GAPDH was used as a loading control. (**G**) BUN, CK, and AST levels were analyzed in the serum at 4 h following LPS (10 mg/kg) injection (*n* = 10/group). PBS was treated as a vehicle negative control for cisplatin in LPS-unstimulated group. A *p* value indicates the significant difference between PBS and LPS. # *p* < 0.05 vs. LPS stimulation. # *p* < 0.05 vs. LPS-injected mice. (**H**) Survival curve (%) was monitored in mice (*n* = 10/group) injected intraperitoneally with LPS (60 mg/kg), *E. coli* (1 × 10^8^ CFU), or TSLP (2 μg) following intravenous injection of cisplatin (100 μg/kg). LPS, lipopolysaccharide; TSLP, thymic stromal lymphopoietin; MDM2, murine double minute 2; BUN, blood urea nitrogen; CK, creatine kinase; AST, aspartate aminotransferase.

In the final series of this study, we sought to determine whether pharmacological inhibition of TSLP level could attenuate a septic response using cisplatin, which is a chemotherapeutic agent against tumor and sepsis. Cisplatin does not have cytotoxicity at doses of 1–100 ng/mL (Figure 6A). Strikingly, as shown in Figure 6B,C, cisplatin treatment resulted in significant reductions in the production (*p* < 0.05) and mRNA expression of TSLP (*p* < 0.05), and expression of MDM2 in LPS-stimulated RAW 264.7 cells. TSLP (*p* < 0.05) and MDM2 levels were suppressed by cisplatin in serum or liver of septic mice (Figure 6D–F), indicating that cisplatin inhibits TSLP level through downregulation of MDM2 expression. Cisplatin significantly decreased BUN, CK, and AST levels in serum of septic mice (*p* < 0.05; Figure 6G). Cisplatin improved the survival rate of the mice following LPS or *E. coli* injection (Figure 6H). However, the recombinant TSLP injection did not affect the survival. 

## 4. Discussion

In this study, we observed that TSLP levels are higher in serum or organ tissues of both mice and humans with sepsis, which functions to induce an inflammatory reaction. In addition, TSLP is produced through the MDM2/NF-κB signaling pathway in LPS-stimulated macrophages. Cisplatin reduces the septic inflammation via down-regulating the TSLP-MDM2 signaling pathway. Taken together, these findings suggest a novel mechanism in that TSLP regulates the development of sepsis. 

Macrophage is a key cell that leads to overwhelming production of cytokines and chemokines, and regulates an intense proinflammatory response during sepsis [36]. Here, we clarified that TSLP is derived from macrophages via TLR4 signaling to LPS or *E. coli*. In addition, TSLP siRNA treatment attenuated the levels of TSLPR and inflammatory cytokines after LPS stimulation in macrophages, suggesting TSLP could regulate the production of inflammatory cytokines via TSLPR as a potential mechanism in LPS-stimulated macrophages. However, the lack of TSLP partially inhibited the production of inflammatory cytokines after LPS stimulation. Thus, we suggest that it would be a TSLP-independent inflammatory response in LPS-stimulated macrophages. Macrophage activation is a pathophysiologic basis for multiple organ dysfunction syndrome (MODS) [37]. Patients with MODS had higher blood levels of AST, ALT, BUN, and CK which are predictive markers of MODS [38]. An increased IL-6 value is the best parameter for predicting development of MODS and mortality [39]. In this study, organ dysfunction was suppressed with lower AST, ALT, BUN, and CK levels in serum of TSLP-deficient mice compared to that of control mice after LPS stimulation. It is noteworthy that recombinant TSLP injection led to a high increase in serum IL-6 level with an increase in AST level after LPS stimulation, although there was no induction of IL-6 by TSLP in the absence of LPS. IL-6 knockout mice showed less hepatic injury by reduced serum ALT levels [40]. High IL-6 level as a marker of disease severity resulted from tissue damage consistent with the concomitantly high AST level [41]. Thus, this study provides evidence that a high TSLP level may lead to organ dysfunction during sepsis, specifically increasing IL-6 and AST levels. This suggests that TSLP produced during sepsis increases IL-6 level, which may induce, at least in part, the changes in AST, ALT, and BUN levels. TSLP deficiency decreased ICAM-1 and MIP2 levels in activated macrophages and septic mice. The elevated TSLP level during sepsis might lead to recruitment of inflammatory cells via ICAM-1 and MIP2. Therefore, we now suggest that TSLP may contribute to organ dysfunction, which is required for sepsis development, affecting inflammatory cell responses. However, we further found that TSLP^−/−^ mice had a liver with lower basal cytokine expression. Ashrin et al. [42] reported that TSLP siRNA injection exhibited a slight decrease in ear skin thickness compared with control siRNA injection in non-stimulated mice. Al-Shami et al. [43] reported that TSLPR knockout mice showed fewer lymphocytes and lower IL-13 level in lung, and lower IgE level in serum compared with wild-type in non-stimulation. Thus, these indicate that the cytokine response could be blunted in the TSLP-deficient mice regardless of stimulus, suggesting that TSLP may be associated with various physiologic and pathologic conditions. Further research is necessary in more experimental models to clarify the roles of TSLP. 

The dose of LPS which leads to death in half of mice (LD50) is about 1–25 mg/kg [44]. In the sepsis experimental model, a high lethal dose of *E. coli* (3 × 10^8^ CFU) [45] or LPS (60 mg/kg; Figure 6H) induced mortality from 2 h or 9 h after injection. To investigate serum TSLP levels at multiple time points during sepsis, low dose of *E. coli* (1 × 10^6^ CFU) or LPS (10 mg/kg) was used in this study. LPS or *E. coli* injection increased serum TSLP levels up to about 1750 pg/mL or 368 pg/mL, respectively. In a cecal ligation and puncture (CLP) model which is a sepsis experimental model, TSLP levels were elevated up to about 70 pg/mL in serum or 200 pg/mL in plasma [8,10]. Thus, we revealed that an increase of TSLP is shown in not only the CLP-induced sepsis model but also the LPS- or *E. coli*-induced sepsis model. 

Kuethe et al. [8] described that TSLP reduces TNF-α production and TSLP blockade results in increased TNF-α levels at the site of infection in the CLP sepsis model. Piliponsky et al. [10] described that TSLP reduces the multiple organ failure that is associated with systemic inflammation, reducing plasma and intraperitoneal levels of proinflammatory cytokines in a CLP sepsis model. However, our results showed that TSLP neutralization allowed significant decreases in production of TNF-α in RAW 264.7 cells; TSLP-deficient mice had significantly less TNF-α levels in liver; nutlin-3a significantly suppressed production of TNF-α and an increase of proinflammatory response by TSLP action in an LPS-induced sepsis model. Even with the same treatment, different experimental models sometimes lead to conflicting results. TNF-α neutralization did not reduce mortality in a CLP sepsis model [46]. On the contrary, TNF-α neutralization reduced mortality in an LPS-induced sepsis model [47]. Our results conflict with the previous reports [8,10]. Thus, we assume that the conflicting results may be, at least in part, due to differences between experimental models such as a CLP model vs. an LPS model.

Moon and Kim demonstrated that TSLP is expressed and produced via NF-κB pathway in mast cells [48]. Jang et al. found that TSLP expression increases through an HIF-1α-dependent mechanism in keratinocytes [49]. NF-κB plays a role in sepsis-associated organ failure [50]. HIF-1α activated by LPS contributed to cytokine activation, symptomatology, and lethality in a LPS-induced sepsis in vivo model [51]. Moreover, MDM2 is a positive activator of both NF-κB and HIF-1α [12,15]. MDM2 was reported to regulate LPS-induced lung dysfunction in mice [52]. Odkhuu et al. reported that LPS enhances activation of NF-κB and production of NO through activation of MDM2 in RAW 264.7 cells [53]. Furthermore, we have now identified that mRNA and protein expressions of MDM2 increase in LPS-stimulated peritoneal macrophages and organs of mice with sepsis. MDM2 upregulated TSLP production via the NF-κB/HIF-1α signaling pathway in activated macrophages. We have further demonstrated that TSLP levels decrease in LPS-stimulated macrophages and mice with sepsis by nutlin-3a, indicating that MDM2 is important in regulating TSLP. Odkhuu et al. [53] reported that LPS stimulation increases phosphorylation of MDM2 in RAW 264.7 cells, suggesting nutlin-3a affects the production of proinflammatory mediators at a late stage after LPS stimulation. Our results showed that serum TSLP levels were reduced by nutlin-3a 4 h after LPS injection. Thus, it is possible that inhibition of MDM2 by nutlin-3a can be suggested to affect an early stage as well as a late stage. In addition, nutlin-3a reduced inflammatory cytokines and CK levels in activated macrophages or septic mice. These observations suggest that TSLP may be produced via the MDM2/NF-κB/HIF-1α signaling pathway in macrophages and contribute to septic responses, further clarifying a regulatory mechanism of TSLP in sepsis as compared with previous reports [8,10]. Therefore, the present findings demonstrate that TSLP can serve as a new target for the development of new drugs for treatment of sepsis.

Here, we found that cisplatin effectively protects against severe sepsis in mice. The study of Ishikawa et al. [54] presents conflicting results with our findings as cisplatin markedly induced an increase in BUN level of LPS-injected mice. However, the concentration of cisplatin utilized in the study of Ishikawa et al. [54] was more than 100-fold higher than that of this study. Interestingly, low-dose cisplatin (1 mg/kg) administration to septic mice improved bacterial clearance and clinical scores [55]. Pan et al. reported that cisplatin decreased the mortality of septic mice at low and nontoxic dose (1 mg/kg) [56]. Furthermore, we have demonstrated that, by using a lower concentration (100 μg/kg) of cisplatin, cisplatin reveals critical pharmacological effects by reducing TSLP levels without cytotoxicity in macrophages and mice during sepsis. A cancer treatment drug, epirubicin also acted therapeutically at a low dose (600 μg/kg) to confer robust protection against severe sepsis in mice [57]. Thus, based on the present results, cisplatin can be considered a good candidate as a useful therapeutic option for patients with sepsis by downregulation of TSLP.

## 5. Conclusions

**Figure 7:**
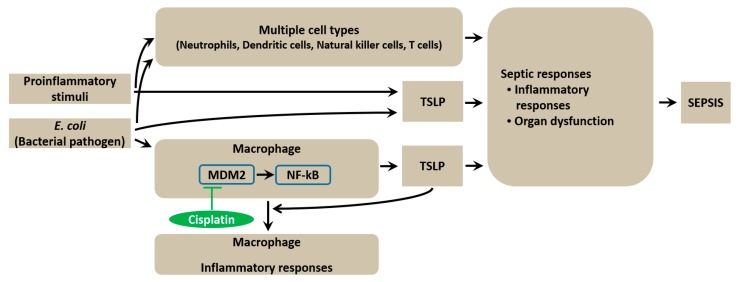
Graphical abstract of TSLP function during sepsis. Production of TSLP is induced via the MDM2/NF-κB signaling pathway in *E. coli*-stimulated macrophages. TSLP affects inflammatory cytokines production and organ dysfunction during sepsis. Cisplatin protects mice against lethal sepsis by inhibiting the series of reactions. TSLP can be induced directly by *E. coli* or other proinflammatory stimuli. Multiple cell types such as neutrophils, dendritic cells, natural killer cells, and T cells might be involved in sepsis.

In summary, this study provides evidence that TSLP is produced via TLR4 signaling in macrophages during sepsis. The MDM2/NF-κB signaling pathway regulates the production of TSLP in *E. coli*-stimulated macrophages. TSLP causes organ dysfunction and an overwhelming systemic inflammatory reaction in septic mice (Figure 7). Therefore, since TSLP regulation could be of therapeutic value for sepsis treatment, understanding the role of TSLP would assist in discovering new targets for sepsis. Establishing TSLP inhibition as a novel sepsis therapy will require a careful assessment of its potential suppressive effects on infectious complications or host defense experimentally and clinically in the future.

## Supplementary Materials

The following are available online at https://www.mdpi.com/2077-0383/8/9/1350/s1, Figure S1: LPS triggers TSLP levels in mice, Figure S2: TSLP mediates inflammatory reactions in septic mice, Figure S3: Systemic inflammatory reaction is blunted in septic TSLP^−/−^ mice, Figure S4: TSLP causes systemic inflammatory reaction and organ dysfunction in septic mice, Figure S5: TSLP is produced in PMA-differentiated THP-1 and HL-60 macrophage-like cells, Figure S6: HIF-1α is involved in LPS-induced TSLP production, Figure S7: MDM2 levels increase in lung, kidney, and large intestine of LPS-injected mice, Figure S8: MDM2 is required for LPS-induced inflammatory responses, Figure S9: Nutlin-3a regulates TSLP levels during sepsis, Figure S10: Nutlin-3a regulates inflammatory responses during sepsis, Figure S11: IL-6 levels are down-regulated in macrophages treated with siRNA for both TSLP and MDM2, Table S1: Comparisons between healthy vs. septic subjects, Table S2: Real-time PCR primers, Table S3: TSLP siRNA sequences.
