# Supplementary material for: TSLP Exacerbates Septic Inflammation via Murine Double Minute 2 (MDM2) Signaling Pathway
Source: J Clin Med. 2019 Sep 1;8(9):1350. doi: 10.3390/jcm8091350 (PMC6780965; doi:10.3390/jcm8091350)
Supplement: Supplementary file 1 [file jcm-08-01350-s001.zip › jcm-554659-supplementary.docx]

****Supplementary Materials****

**TSLP Exacerbates Septic Inflammation via MDM2 Signaling Pathway**

**Na-Ra Han^1,†^, Phil-Dong Moon^1,2,†^, Hyung-Min Kim^1,*^ and Hyun-Ja Jeong^3,*^**

^1^ Department of Pharmacology, College of Korean Medicine, Kyung Hee University, Seoul 02447, Republic of Korea; nrhan@khu.ac.kr (N.-R.H.); pdmoon@khu.ac.kr (P.-D.M.)

^2^ Center for Converging Humanities, Kyung Hee University, Seoul 02447, Republic of Korea

^3^ Department of Food Science & Technology, Inflammatory Diseases Research Center, Hoseo University, Asan, Chungnam 31499, Republic of Korea

^*^ Correspondence: hmkim@khu.ac.kr (H.-M.K.); hjjeong@hoseo.edu (H.-J.J.);

Tel.: +82-2-961-9448 (H.-M.K.); +82- 41-540-9681 (H.-J.J.)

^†^ These authors contributed equally to this work.

**Supplementary Figures**


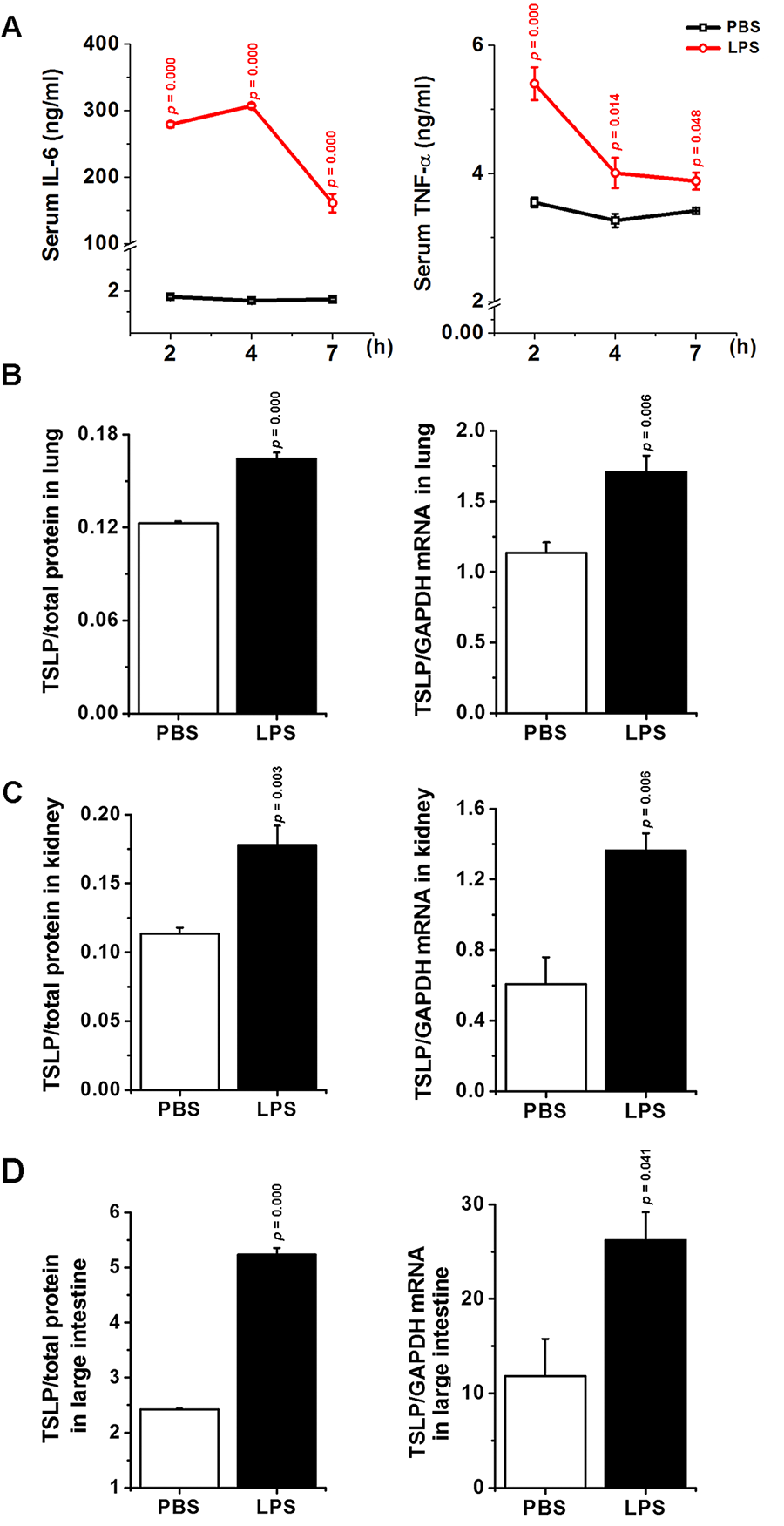


****Figure S1.** LPS triggers TSLP levels in mice. (**A**) IL-6 and TNF-ɑ levels in serum after LPS (10 mg/kg) injection were analyzed by ELISA. (Left) Protein and (right) mRNA expressions of TSLP in (**B**) lung, (**C**) kidney, and (**D**) large intestine of mice 12h after LPS (10 mg/kg) injection were analyzed by ELISA and real-time PCR. Adducts were normalized to total protein in each homogenate for ELISA analysis. For PCR analysis, results were normalized to GAPDH. (*n* = 10/group). A *p* value indicates the significant difference between PBS and LPS.**


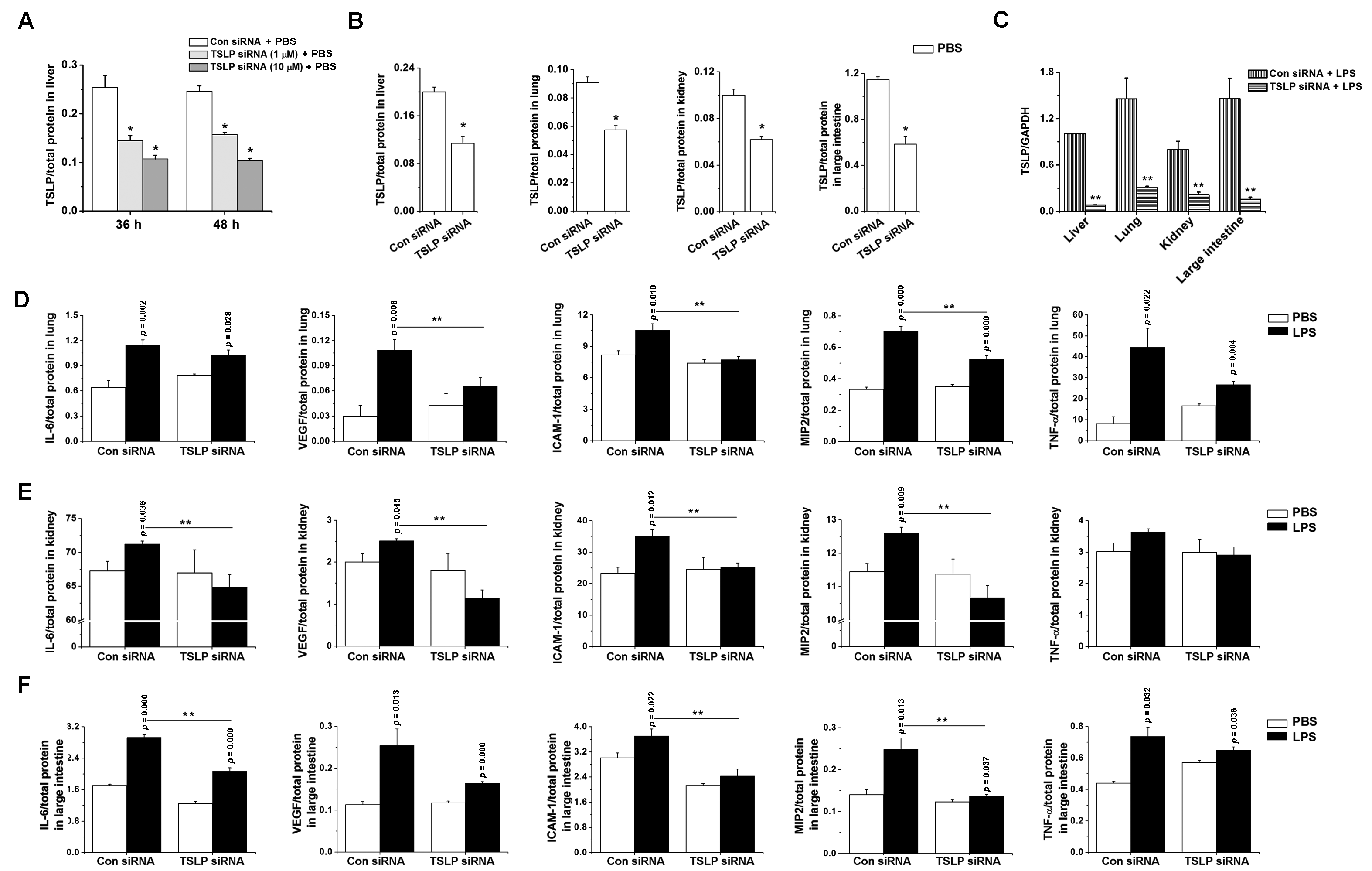


****Figure S2.** TSLP mediates inflammatory reactions in septic mice. (**A**)** Mice were intravenously injected with scramble control siRNA or TSLP**-specific** siRNA mixture (**1 µM or 10 µM)** 24 h before PBS injection. The mice were euthanized 12 h or 24 h after PBS injection. **TSLP levels in liver** homogenates **36 h or 48 h after** TSLP**-specific** siRNA mixture injection **were analyzed by ELISA. (**B**) TSLP levels in liver, lung, kidney, and large intestine** homogenates **36 h after** TSLP**-specific** siRNA mixture (**10 µM**) injection **were analyzed by ELISA. ^*^*p <* 0.05 vs Con siRNA-**received **mice. (**C**) M**ice were injected with TSLP**-specific** siRNA mixture (**10 µM)** 24 h before LPS injection (10 mg/kg). **TSLP mRNA levels in liver, lung, kidney, and large intestine** homogenates **obtained 12 h after LPS injection were analyzed by real-time** PCR**. Each level in (**D**) lung, (**E**) kidney, and (**F**) large intestine** homogenates **12h after LPS (10 mg/kg) injection was analyzed by ELISA. Adducts were normalized to total protein in each homogenate. (*n* = 10/group).** **A *p* value indicates the significant difference between PBS and LPS. ^**^*p <* 0.05 vs Con siRNA-received and LPS-injected control** mice**. Abbreviation: PBS, phosphate-buffered saline; LPS, lipopolysaccharide; Con, control; siRNA, small interfering RNA; VEGF, vascular endothelial growth factor; ICAM-1, intercellular adhesion molecule-1; MIP2, macrophage inflammatory protein 2.**


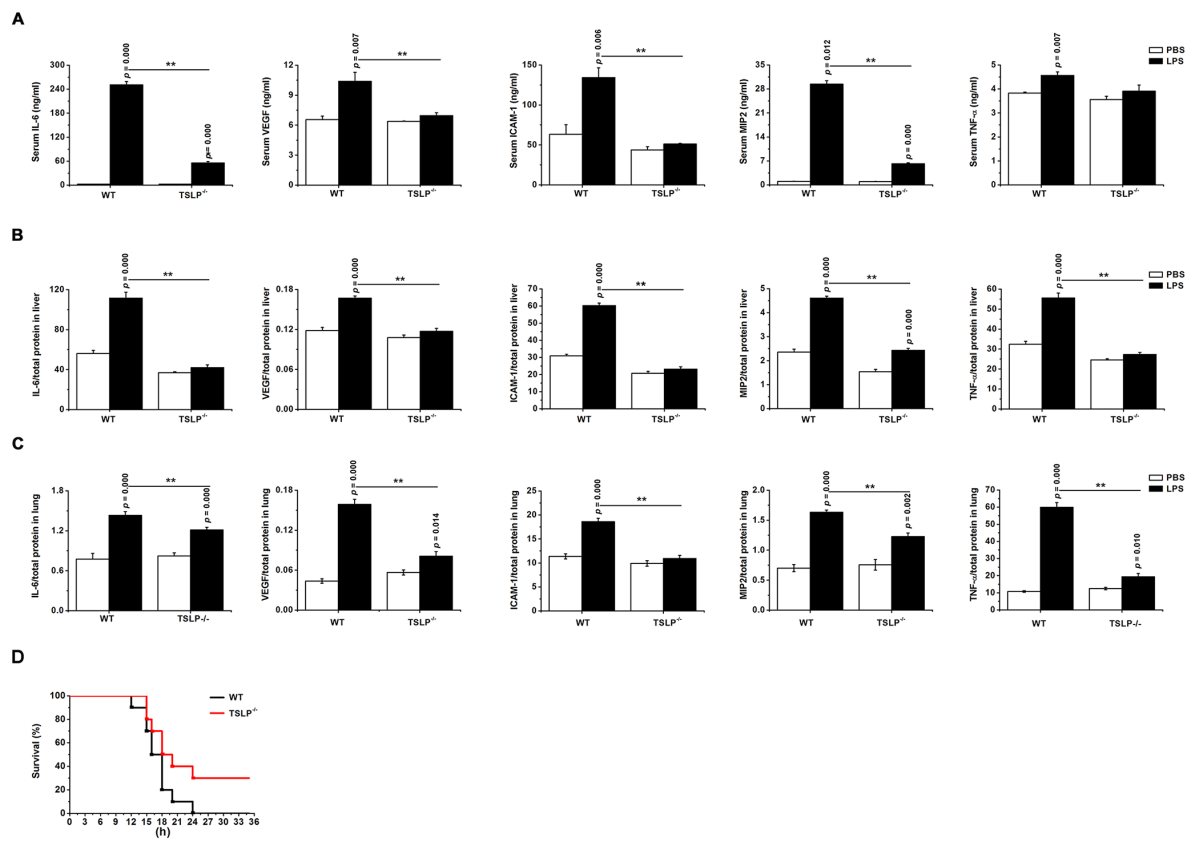


****Figure S3.** Systemic inflammatory reaction is blunted in septic TSLP^-/-^ mice.** **TSLP levels were analyzed by ELISA in (**A**) serum 4 h, (**B**)** liver and **(**C**) lung** homogenate **12 h after LPS injection** (10 mg/kg) in **TSLP^-/-^ mice. Adducts were normalized to total protein in homogenate. (*n* = 5/group). A *p* value indicates the significant difference between PBS and LPS. ^**^*p <* 0.05 vs LPS-injected WT mice. (**D**) Survival curve (%) was monitored in mice (*n* = 10/group) injected intraperitoneally with a lethal dose of LPS (60 mg/kg). Abbreviation: WT, wild-type; PBS, phosphate-buffered saline; LPS, lipopolysaccharide; VEGF, vascular endothelial growth factor; ICAM-1, intercellular adhesion molecule-1; MIP2, macrophage inflammatory protein 2.**


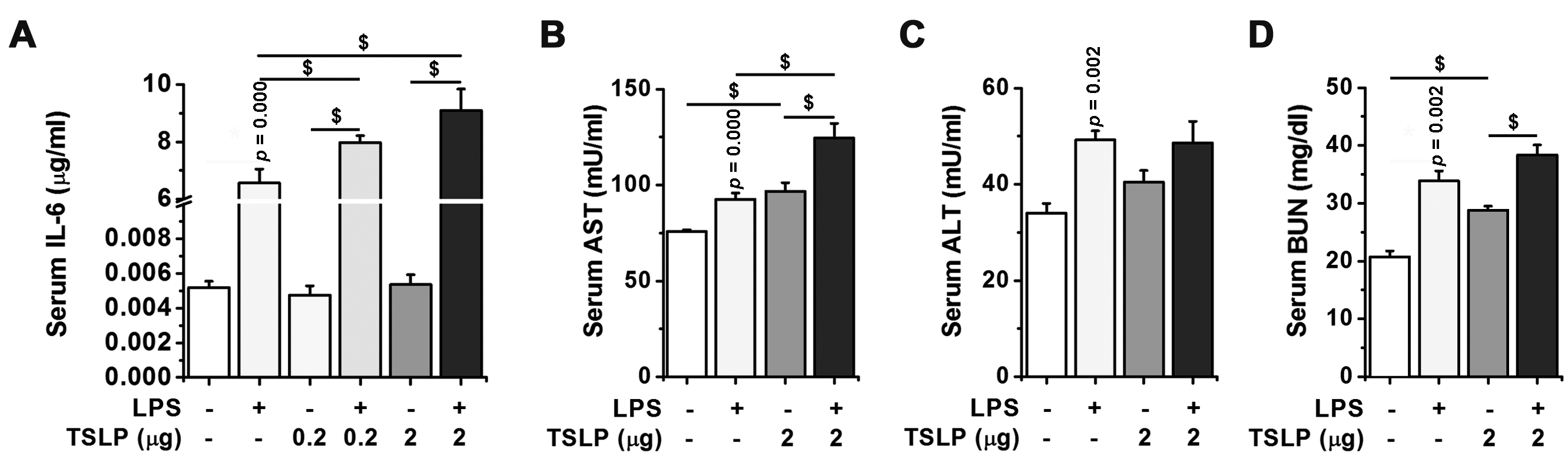


****Figure S4.**** TSLP causes systemic inflammatory reaction and organ dysfunction in septic mice. **Each level was analyzed in the serum at 4 h following** injection with recombinant mouse TSLP (0.2 or 2 μg) or LPS (10 mg/kg). **(*n* = 10/group). A *p* value indicates the significant difference between PBS and LPS. ^$^*p <* 0.05. Abbreviation: LPS, lipopolysaccharide; AST, aspartate aminotransferase; ALT, alanine aminotransferase, BUN, blood urea nitrogen.**


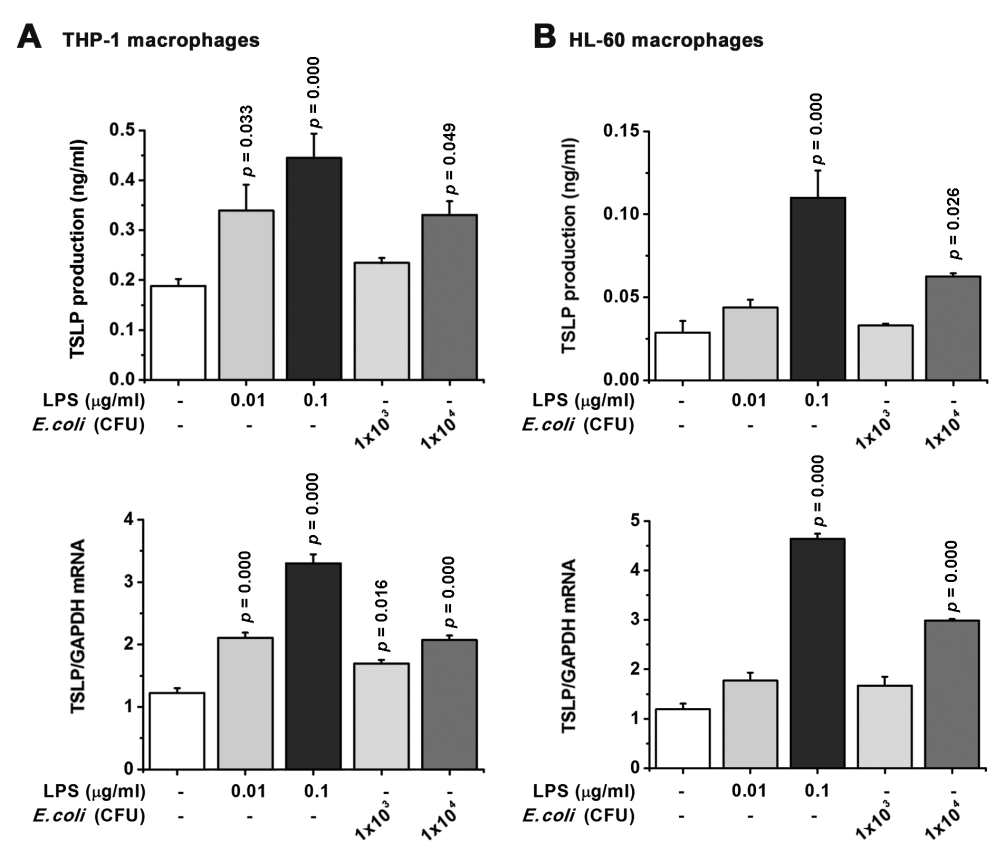


****Figure S5.** TSLP is produced in PMA-differentiated THP-1 and** HL-60 macrophage-like cells**. (**A,B upper panel**) PMA-differentiated THP-1 and** HL-60 **cells were stimulated with LPS or *E. coli* for 24 h. The production of TSLP was detected by ELISA. (**A,B lower panel**) PMA-differentiated THP-1 and** HL-60 **cells were stimulated with LPS or *E. coli* for 8 h. The mRNA expression of TSLP was detected by real-time PCR. Data are representative of three independent experiments (*n* = 5/group). A *p* value indicates the significant difference between PBS and LPS. Abbreviation: LPS, lipopolysaccharide; *E.coli, Escherichia coli.***


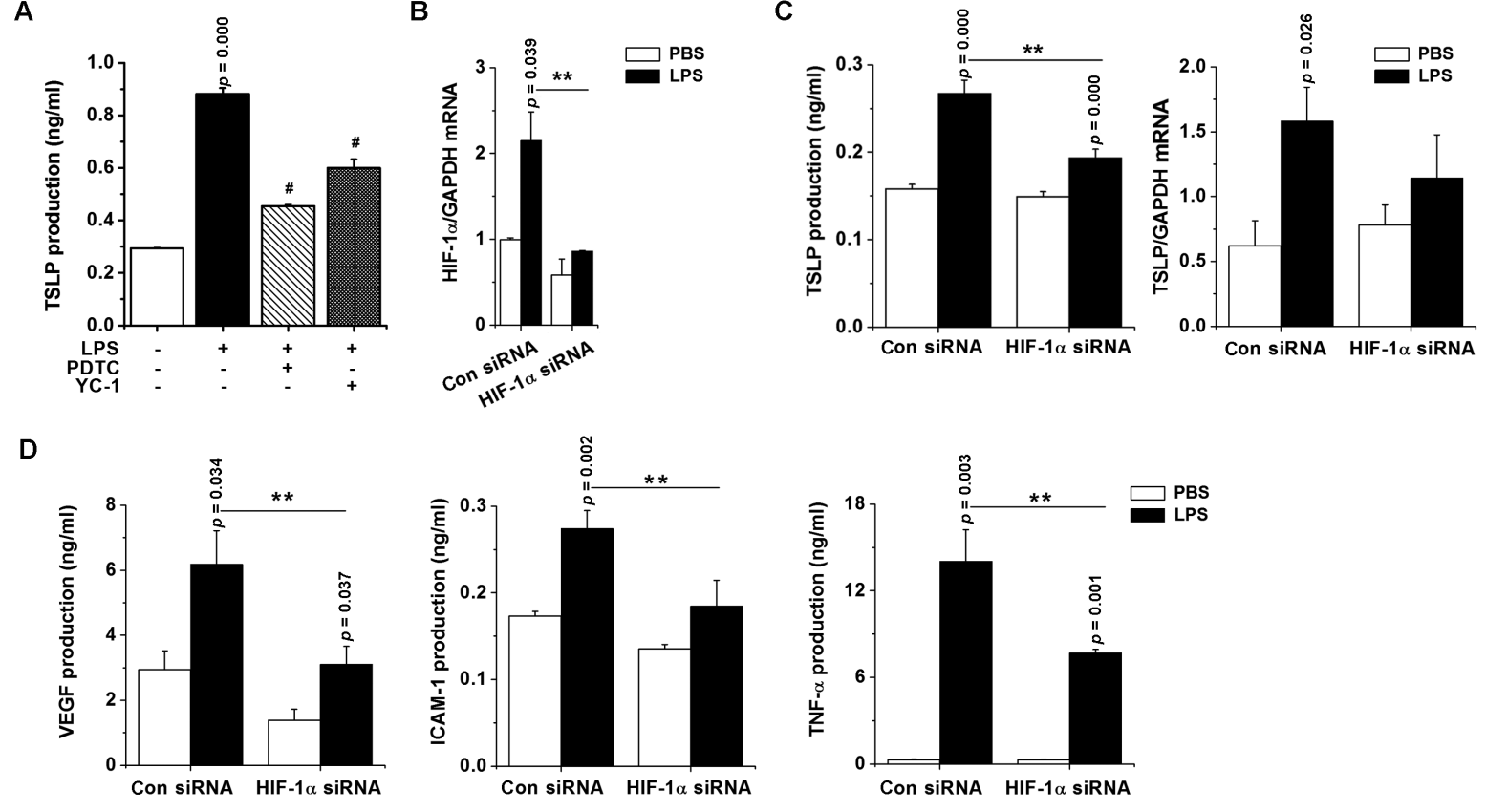


****Figure S6.** HIF-1α is involved in LPS-induced TSLP production. (**A**) RAW 264.7 cells were pretreated with PDTC (**1 μM**) or YC-1 (**10 μM**) 2 h prior to incubation of LPS (0.1 μg/ml) for 24 h. TSLP production was analyzed by ELISA. ^#^*p <* 0.05 vs LPS stimulation. (**B**) RAW 264.7 cells were transfected with scramble control siRNA or HIF-1α-specific siRNA. After LPS** stimulation**, HIF-1α mRNA expression was analyzed by real-time PCR. (**C, left**) TSLP production 24 h after LPS stimulation was analyzed in the transfected cells by ELISA. (**C, right**) TSLP mRNA expression 8 h after LPS stimulation was analyzed in the transfected cells by real-time PCR. (**D**) Each level 24 h after LPS stimulation from the transfected cells was analyzed by ELISA. Data are representative of three independent experiments (*n* = 5/group). A *p* value indicates the significant difference between PBS and LPS. ^**^*p <* 0.05 vs Con siRNA transfection and LPS stimulation. Abbreviation:** PDTC, pyrrolidine dithiocarbamate; YC-1, 3-(5′-hydroxy-methyl-2′-furyl)-1-benzylindazole; **HIF-1α, hypoxia-inducible factor-1α; Con, control; siRNA, small interfering RNA.**


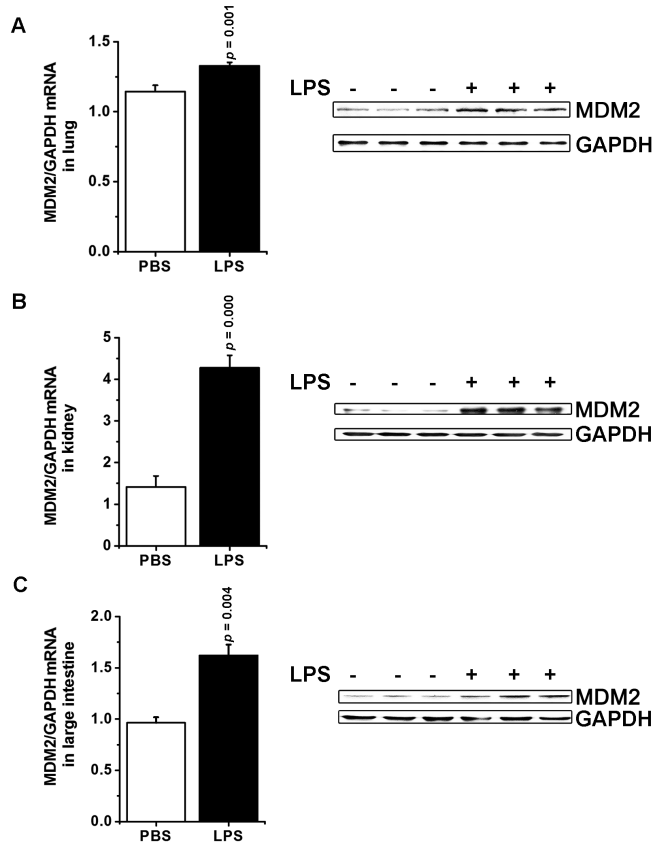


****Figure S7.** MDM2 levels increase in lung, kidney, and large intestine of LPS-injected mice. (Left) The mRNA and (right) protein expression of MDM2 were analyzed in (**A**) lung, (**B**) kidney, (**C**) large intestine of mice 12h after LPS (**10 mg/kg**) injection by real-time PCR and Western blot. For PCR analysis, results were normalized to GAPDH. For immunoblots, GAPDH was used as a loading control. (*n* = 10/group). A *p* value indicates the significant difference between PBS and LPS. Abbreviation: PBS, phosphate-buffered saline; LPS, lipopolysaccharide; MDM2, murine double minute 2.**


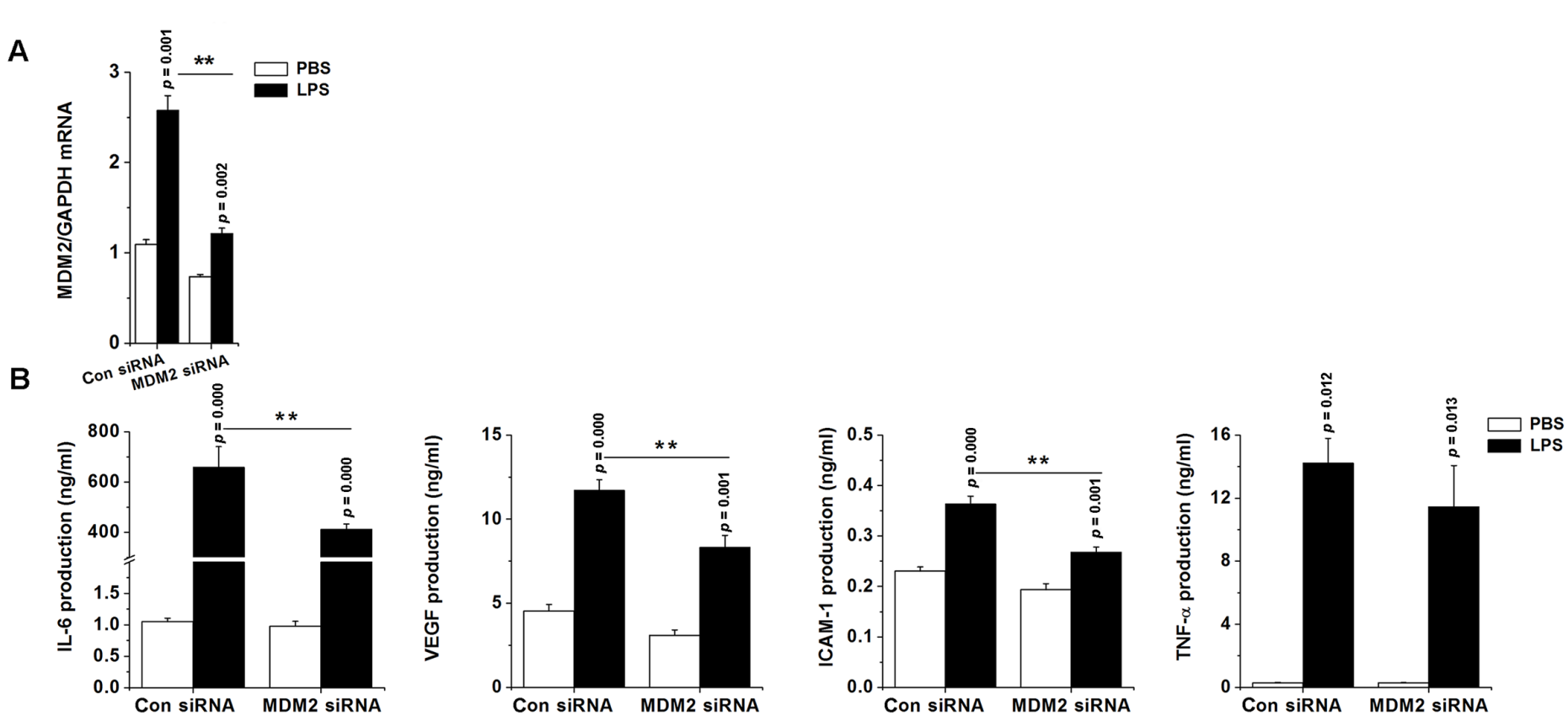


****Figure S8.** MDM2 is required for LPS-induced inflammatory responses. (**A**) RAW 264.7 cells were transfected with scramble control siRNA or MDM2-specific siRNA. After LPS (0.1 μg/ml)** stimulation**, MDM2 mRNA expression was analyzed by real-time PCR. (**B**) Each production from MDM2**-specific **siRNA-transfected RAW 264.7 cells 24 h after LPS stimulation was analyzed by ELISA. Data are representative of three independent experiments (*n* = 5/group). A *p* value indicates the significant difference between PBS and LPS. ^**^*p <* 0.05 vs Con siRNA transfection and LPS stimulation. Abbreviation: PBS, phosphate-buffered saline; LPS, lipopolysaccharide; MDM2, murine double minute 2; Con, control; siRNA, small interfering RNA.**


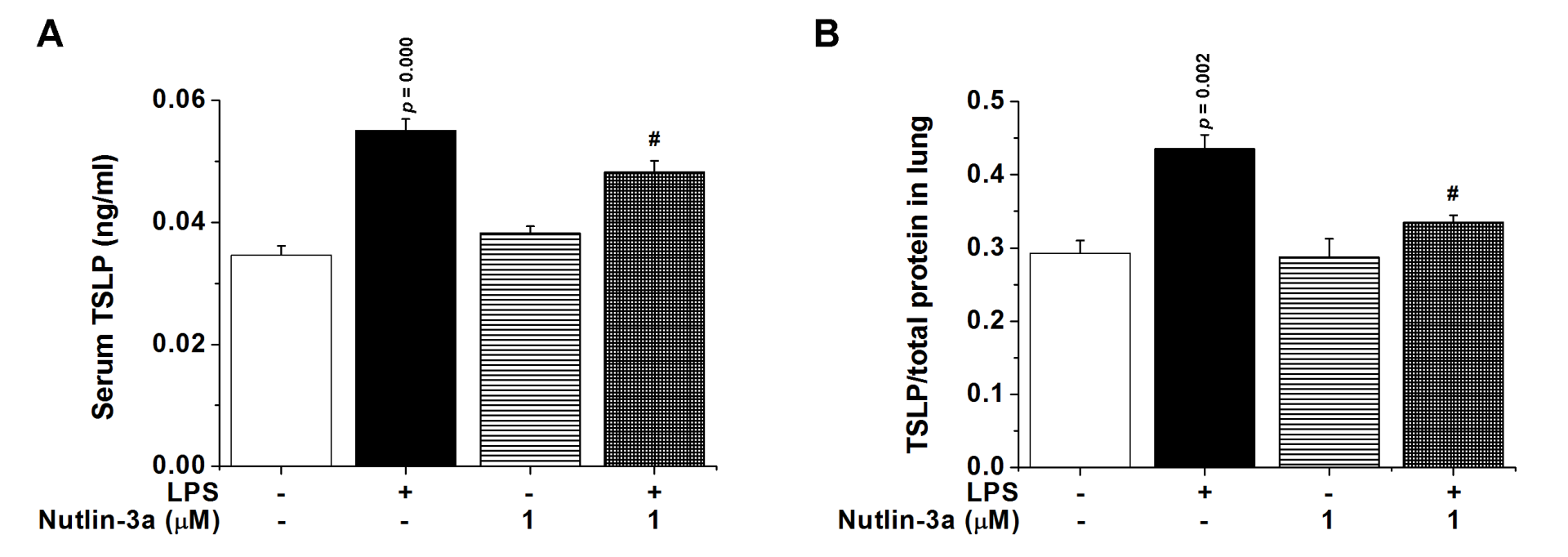


****Figure S9.**** Nutlin-3a regulates TSLP levels during sepsis. **(**A**)** **Serum at 24 h and (**B**) lung homogenate at 12 h following LPS injection from nutlin-3a-treated septic mice were subjected to ELISA. (*n* = 10/group). Adducts were normalized to total protein in lung homogenate. A *p* value indicates the significant difference between PBS and LPS. ^#^*p* < 0.05 vs LPS-injected mice.**


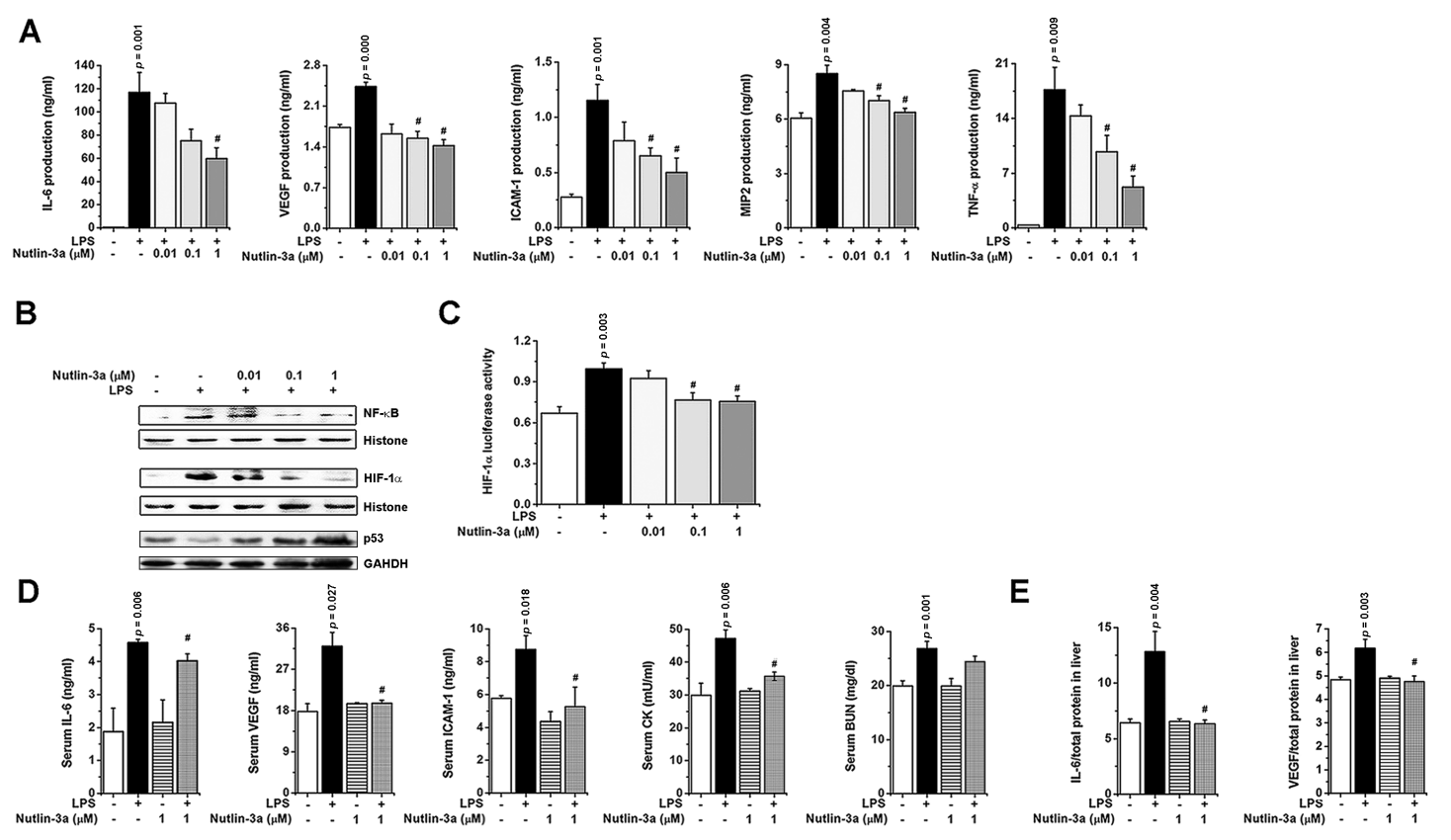


****Figure S10.**** Nutlin-3a regulates inflammatory responses during sepsis. **(**A**) RAW 264.7 cells were pretreated with nutlin-3a, followed by incubation with LPS (0.1 μg/ml) for 24 h. Each level was measured by ELISA. (**B**) NF-kB, HIF-1α, and p53 expressions were determined in nuclear faction of RAW 264.7 cells by Western blot. For immunoblots, histone was used as a loading control. (**C**) HIF-1α activity was measured with a luciferase assay. Data are representative of three independent experiments (*n* = 5/group). ^#^*p <* 0.05 vs LPS-stimulated cells. (**D**) Serum at 4 h and (**E**)** liver homogenate **at 12 h following LPS** (10 mg/kg) **injection from** nutlin-3a-treated septic mice were subjected to ELISA. **Adducts were normalized to total protein in** liver **homogenate. (*n* = 10/group). A *p* value indicates the significant difference between PBS and LPS. ^#^*p <* 0.05 vs LPS-injected** mice**. Abbreviation: LPS, lipopolysaccharide; VEGF, vascular endothelial growth factor; ICAM-1, intercellular adhesion molecule-1; MIP2, macrophage inflammatory protein 2; HIF-1α, hypoxia-inducible factor-1α; CK, creatine kinase; BUN, blood urea nitrogen.**


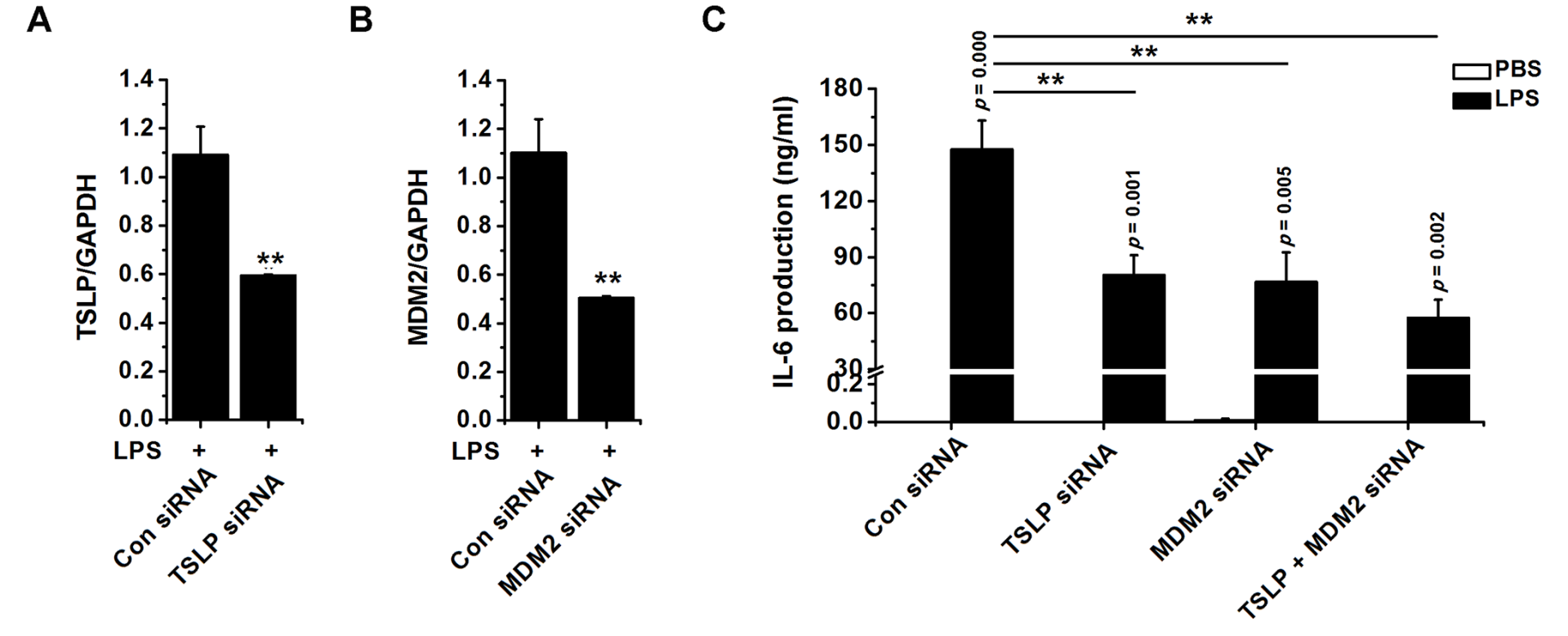


****Figure S11.**** IL-6 levels are down-regulated in macrophages treated with siRNA for both TSLP and MDM2. RAW 264.7 cells were transfected with scramble control siRNA, TSLP-specific siRNA, MDM2-specific siRNA, or siRNA for both TSLP and MDM2. After LPS stimulation, (**A**) TSLP and (**B**) MDM2 mRNA levels were analyzed in the transfected cells by real-time PCR. (**C**) IL-6 **production 24 h after LPS stimulation was analyzed by ELISA. A *p* value indicates the significant difference between PBS and LPS.** ^**^*p* < 0.05 vs Con siRNA transfection and LPS stimulation. **Abbreviation: Con, control; siRNA, small interfering RNA; MDM2, murine double minute 2.**

****Table S1.** Comparisons between healthy vs septic subjects.**

| **Characteristics** | **Healthy (*n* = 20)** | **Septic (*n* = 30)** | |
| --- | --- | --- | --- |
|  |  | **Survival (*n* = 19)** | **Non-survival (*n* = 11)** |
| Age | 60.20 ± 1.97 | 35.83 ± 8.99 | 67.91 ± 3.31 |
| Sex | F = 10 / M = 10 | F = 7 / M = 12 | F = 3 / M = 8 |
| Race | All Mongoloid | All Mongoloid | All Mongoloid |
| WBC (×10^3^/mm^3^) | 7.01 ± 0.30 | 15.45 ± 1.15  (*p* = 0.001, vs Healthy) | 18.08 ± 3.60  (*p* = 0.012, vs Healthy) |
| Platelet (×10^3^/mm^3^) | 263.30 ± 11.71 | 273.84 ± 26.59 | 116.00 ± 24.87  (*p* = 0.001, vs Healthy)  (*p* = 0.001, vs Survival) |
| HR (beats/min) | - | 126.90 ± 10.50 | 87.55 ± 5.87  (*p* = 0.003, vs Survival) |
| RR (breaths/min) | - | 35.95 ± 4.53 | 19.91 ± 0.74  (*p* = 0.002, vs Survival) |
| BT (ºC) | - | 37.40 ± 0.27 | 36.55 ± 0.22  (*p* = 0.022, vs Survival) |
| Infection source | - | *Escherichia coli* = 3  *Staphylococcus aureus* = 3  *Staphylococcus epidermidis* = 2  *Streptococcus agalactiae* = 2  *Streptococcus pneumonia* = 2  *Coagulase negative Staphylococcus* = 1  *Enterobacter cloacae* = 1  *Enterococcus faecalis* = 1  *Enterococcus faecium* = 1  *Klebsiella pneumoniae ssp pneumonia* = 1  *Streptococcus aureus* = 1  *Viridans streptococcus group* = 1 | *Escherichia coli* = 4  *Staphylococcus aureus* = 2  *Acinetobacter baumannii* = 1  *Klebsiella pneumoniae ssp pneumonia* = 1  *Pseudomonas aeruginosa* = 1  *Streptococcus agalactiae*=1  *Streptococcus pneumonia* = 1 |
| INR | - | 1.12 ± 0.07 | 1.68 ± 0.15  (*p* = 0.005, vs Survival) |
| aPTT (sec) | - | 54.17 ± 7.68 | 55.88 ± 9.35 |
| Total Bilirubin (mg/dl) | - | 5.41 ± 1.92 | 7.33 ± 2.57 |
| Creatinine (mg/dl) | - | 1.13 ± 0.31 | 1.43 ± 0.21 |
| C-reactive protein (mg/l) | - | 70.32 ± 20.98 | 184.53 ± 28.71  (*p* = 0.003, vs Survival) |
| SBP (mmHg) | - | 97.79 ± 5.77 | 100.82 ± 4.38 |
| DBP (mmHg) | - | 61.16 ± 3.25 | 61.91 ± 2.92 |

**F, female; M, male; WBC, white blood cell; HR, heart rate; RR, respiratory rate; BT, body temperature; INR, international normalized ratio ; aPTT, activated partial thromboplastin time; SBP, systolic blood pressure ; DBP, diastolic blood pressure.**

****Table S2.** Real-time PCR primers.**

| **Species** | **Target gene** | **Forward** | **Reverse** |
| --- | --- | --- | --- |
| Mouse | TSLP | 5′-AGAGAAGCCCTCAATGACCA-3′ | 5′- TCTGGAGATTGCATGAAGGA-3′ |
|  | IL-6 | 5′-CCAGAAACCGCTATGAAGTTCCT-3′ | 5′-CACCAGCATCAGTCCCAAGA-3′ |
|  | VEGF | 5′-ACCGCCTTGGCTTGTCACAT-3′ | 5′-ACCGCGAGGCAGCTTGAGTTA-3′ |
|  | ICAM-1 | 5′-TACGTGTGCCATGCCTTTAGC-3′ | 5′-GCCCACAATGACCAGCAGTA-3′ |
|  | MIP2 | 5′-AGTGAACTGCGCTGTCAATGC-3′ | 5′-AGGCAAACTTTTTGACCGCC-3′ |
|  | TNF-α | 5′-CAGACCCTCACACTCAGATCATCT-3′ | 5′-CCTCCACTTGGTGGTTTGCTA-3′ |
|  | HIF-1α | 5′-GAAGTGGCTTTGGAGTTTCCG-3′ | 5′-CATCTCTCTGGATTTTGGCAGC-3′ |
|  | MDM2 | 5′-AAGATCCTGATGCGAGGGCGTC-3′ | 5′-TTGATCCGAGCCTGGGTCTGTG-3′ |
|  | TSLPR | 5′-GGGCCATGGTGTTTAAGGCTA-3′ | 5′-GAGCAGCGTCACATTCCAAG-3′ |
|  | IL-7Rɑ | 5′-CAGAAATAGGCCTCCTGTGTATCAA-3′ | 5′-GGATTCCCGACTGGAAAGG-3′ |
|  | GAPDH | 5′-GGCAAATTCAACGGCACA-3′ | 5′- GTTAGTGGGGTCTCGCTCCTG-3′ |
| Human | TSLP | 5′-CCCAGGCTATTCGGAAACTCAG-3′ | 5′-CGCCACAATCCTTGTAATTGTG-3′ |
|  | GAPDH | 5′-TCGACAGTCAGCCGCATCTTCTTT-3′ | 5′-ACCAAATCCGTTGACTCCGACCTT-3′ |

****Table S3.** TSLP siRNA sequences**

|  | **Sequences** |
| --- | --- |
| siRNA1 | CAGUAUAGGUGCUUUAAAU |
| siRNA2 | UGAGAGAAAUGACGGUACU |
| siRNA3 | GAAAAUGACAGUUCGGGCA |
| siRNA4 | AAUGAGCAAUAGACCGUUA |
